# Molecular Dating of the Emergence of Anaerobic Rumen Fungi and the Impact of Laterally Acquired Genes

**DOI:** 10.1128/mSystems.00247-19

**Published:** 2019-08-27

**Authors:** Yan Wang, Noha H. Youssef, Matthew Brian Couger, Radwa A. Hanafy, Mostafa S. Elshahed, Jason E. Stajich

**Affiliations:** a Department of Microbiology and Plant Pathology, University of California—Riverside, Riverside, California, USA; b Institute for Integrative Genome Biology, University of California—Riverside, Riverside, California, USA; c Department of Microbiology and Molecular Genetics, Oklahoma State University, Stillwater, Oklahoma, USA; d High Performance Computing Center, Oklahoma State University, Stillwater, Oklahoma, USA; Dartmouth College

**Keywords:** comparative genomics, divergence time estimation, evolution, HGT, phylogenomics

## Abstract

The anaerobic gut fungi (AGF), or Neocallimastigomycota, inhabit the rumen and alimentary tract of herbivorous mammals, where they play important roles in the degradation of plant fiber. Comparative genomic and phylogenomic analyses of the AGF have long been hampered by their fastidious growth condition, as well as their large (up to 200 Mb) and AT-biased (78 to 84%) genomes. We sequenced 21 AGF transcriptomes and combined them with 5 available AGF genome sequences to explore their evolutionary relationships, time their divergence, and characterize gene gain/loss patterns associated with their evolution. We estimate that the most recent common ancestor of the AGF diverged 66 (±10) million years ago, a time frame that coincides with the evolution of grasses (Poaceae), as well as the mammalian transition from insectivory to herbivory. The concordance of independent estimations suggests that AGF have been important in shaping the success of mammalian herbivory transition by improving the efficiency of energy acquisition from recalcitrant plant materials. Comparative genomics identified multiple lineage-specific genes in the AGF, two of which were acquired from rumen gut bacteria and animal hosts via horizontal gene transfer (HGT). A third AGF domain, plant-like polysaccharide lyase, represents a novel gene in fungi that potentially aids AGF to degrade pectin. Analysis of genomic and transcriptomic sequences confirmed both the presence and expression of these lineage-specific genes in nearly all AGF clades. These genetic elements may contribute to the exceptional abilities of AGF to degrade plant biomass and enable metabolism of the rumen microbes and animal hosts.

**IMPORTANCE** Anaerobic fungi living in the rumen of herbivorous mammals possess an extraordinary ability to degrade plant biomass. We examined the origin and genomic composition of these poorly characterized anaerobic gut fungi using both transcriptome and genomic data. Phylogenomics and molecular dating analyses found remarkable concurrence of the divergence times of the rumen fungi, the forage grasses, and the dietary shift of ancestral mammals from primarily insectivory to herbivory. Comparative genomics identified unique machinery in these fungi to utilize plant polysaccharides. The rumen fungi were also identified with the ability to code for three protein domains with putative functions in plant pectin degradation and microbial defense, which were absent from all other fungal organisms (examined over 1,000 fungal genomes). Two of these domains were likely acquired from rumen gut bacteria and animal hosts separately via horizontal gene transfer. The third one is a plant-like polysaccharide lyase, representing a unique fungal enzyme with potential pectin breakdown abilities.

## Abstract (precis)

Anaerobic fungi living in the rumen of herbivorous mammals possess an extraordinary ability to degrade plant biomass. We examined the origin and genomic composition of these poorly characterized anaerobic gut fungi using both transcriptome and genomic data. Phylogenomics and molecular dating analyses found remarkable concurrence of the divergence times of the rumen fungi, the forage grasses, and the dietary shift of ancestral mammals from primarily insectivory to herbivory. Comparative genomics identified unique machinery in these fungi to utilize plant polysaccharides. The rumen fungi were also identified with the ability to code for three protein domains with putative functions in plant pectin degradation and microbial defense, which were absent from all other fungal organisms (examined over 1,000 fungal genomes). Two of these domains were likely acquired from rumen gut bacteria and animal hosts separately via horizontal gene transfer. The third one is a plant-like polysaccharide lyase, representing a unique fungal enzyme with potential pectin breakdown abilities.

## INTRODUCTION

Diverse microbes inhabit the digestive tract of ruminant mammals and contribute to the degradation of ingested plant fibers, a process that liberates nutrients for their hosts. Large-scale genomic and metagenomic sequencing of rumen microbes have produced hundreds of novel bacterial genomes, enabling the discovery of plant biomass-degrading enzymes and patterns of genomic evolution (1, 2). However, eukaryotic members of the rumen microbial community have been less intensely studied (3, 4). Members of the phylum Neocallimastigomycota (anaerobic gut fungi [AGF]) are important members of the rumen and hindgut of a wide range of herbivorous mammals and reptiles (5). To survive in this anoxic and prokaryote-dominated environment, extant AGF members have undergone multiple structures and metabolic adaptations, including the loss of the mitochondria, gain of a hydrogenosome, loss of respiratory capacities, and substitution of ergosterol with tetrahymanol in the cell membrane (6). Importantly, all known AGF taxa have a remarkably efficient plant biomass degradation machinery, which may be critical for competing with other microbes for resources and establishing growth in the herbivorous gut. Such capacity is reflected in the possession of an impressive arsenal of plant biomass degradation enzymes and the production of the cellulosomes, extracellular structures that harbor multiple enzymes bound to scaffoldins (4). These metabolic and structural adaptations improve the survivability, fitness, and competitiveness of the AGF in the herbivorous gut, but the genetic and evolutionary origins of these changes remain largely undescribed (3, 7). Previous genomic investigations of the AGF have identified a massive number of carbohydrate-active enzymes encoded by genes with foreign origins, presumably from multiple lineages of bacteria through independent horizontal gene transfer (HGT) events (3, 4, 7). Recently, hundreds of HGT elements were detected in AGF and are suggested to have enabled the fungi to expand their substrate utilization range, augment their biosynthetic capabilities, and shape a phylogenetically distinct fungal lineage (8). In fact, HGT examples from bacteria to fungi have been documented extensively (9–12). However, HGT elements in fungi that have been transferred from other eukaryotes are still rare, with only a few described cases from animals (13), oomycetes (14), or plants (15). The rumen is an intriguing context to explore patterns of HGT, where DNA and RNA are liberated when cells are disrupted by degradative enzymes. Competing organisms can find an advantage by acquiring foreign genes that operate efficiently in an anaerobic environment to obtain nutrients from recalcitrant plant fibers or to recognize other microbes. Our study took a conservative approach to identify candidate HGT by focusing on protein domains rather than entire genes and focusing on instances that appear to be unique gains in the AGF and that are missing in all other fungal lineages.

The Neocallimastigomycota are classified within the Chytridiomycota (chytrid) fungi, which share the trait of a flagellated zoospore stage (16–19). Efforts to resolve the phylogenetic relationship of AGF and their sister lineages using ribosomal markers have yielded conflicting topologies (20, 21). A multilocus phylogeny is needed to evaluate their evolutionary relationships and to estimate the divergence time of the AGF. Using genomes and transcriptomes from 26 different AGF taxa (Table 1) covering seven out of the 10 recognized genera, we reconstructed a robust phylogenomic tree of the AGF and estimated their divergence time. We compared the genomes or transcriptomes of AGF and their non-rumen-associated relatives in Chytridiomycota to identify unique and shared genome contents. This study examined the relatively recent divergence of the AGF clade and revealed a concordance of the divergence time of the Neocallimastigomycota fungi with both the mammalian host transition to herbivory and the diversification events of the forage grasses. As the AGF are well known for their exceptional efficiency at plant biomass degradation, we also explored the diverse genetic components of these fungi. We discovered two potential HGT elements that were found to be unique to the AGF, which are predicted to have originated from animals or bacteria. Examination of the family of bacterial transferred genes revealed multiple intron insertion events that occurred after the HGT acquisition process, which are present in all five AGF genomes. Comparative analyses of these genes suggest putative intron insert events involved the intragenic duplication of coding sequences. In addition, a novel plant polysaccharide lyase was revealed from both AGF genomes and transcriptomes that has never been reported from any known fungal genomes or genetic studies. The evolutionary genomic investigation of these rumen-inhabiting fungi provides perspective on the concordant timing of their divergence with the ecological niche they inhabit and the potential role of HGT in the accumulation of lineage-specific processes that may contribute to their unique biology.

**TABLE 1:** Information for the AGF strains included in this study

Organism	Strain	Accession no.	Type	Host	Reference or source
*Anaeromyces contortous*	Na	GGWN00000000	Transcriptome	Cow	This study
*Anaeromyces contortous*	C3J	GGWO00000000	Transcriptome	Cow	This study
*Anaeromyces contortous*	G3G	GGWR00000000	Transcriptome	Goat	This study
*Anaeromyces contortous*	O2	GGWQ00000000	Transcriptome	Cow	This study
*Anaeromyces contortous*	C3G	GGWR00000000	Transcriptome	Cow	This study
*Anaeromyces robustus*	S4	MCFG00000000	Genome	Sheep	4
*Caecomyces* sp.	Brit4	GGWS00000000	Transcriptome	Cow	This study
*Caecomyces* sp.	Iso3	GGXE00000000	Transcriptome	Cow	This study
*Feramyces austinii*	WSF3a	GGWU00000000	Transcriptome	Aoudad	This study
*Feramyces austinii*	WSF2c	GGWT00000000	Transcriptome	Aoudad	This study
*Orpinomyces* sp.	D3A	GGWV00000000	Transcriptome	Cow	This study
*Orpinomyces* sp.	D3B	GGWW00000000	Transcriptome	Cow	This study
*Orpinomyces* sp.	D4C	GGWX00000000	Transcriptome	Cow	This study
*Pecoramyces ruminantium*	C1A	ASRE00000000	Genome	Cow	3
*Pecoramyces* sp.	S4B	GGWY00000000	Transcriptome	Sheep	This study
*Pecoramyces* sp.	FX4B	GGWZ00000000	Transcriptome	Cow	This study
*Pecoramyces* sp.	FS3c	GGXF00000000	Transcriptome	Cow	This study
*Pecoramyces* sp.	YC3	GGXA00000000	Transcriptome	Cow	This study
*Piromyces finnis*	Pirfi3	MCFH00000000	Genome	Horse	4
*Piromyces* sp.	E2	MCNC00000000	Genome	Elephant	4
*Piromyces* sp.	A1	GGXB00000000	Transcriptome	Sheep	This study
*Piromyces* sp.	B4	GGXH00000000	Transcriptome	Cow	This study
*Piromyces* sp.	B5	GGXI00000000	Transcriptome	Cow	This study
*Neocallimastix californiae*	G1	MCOG00000000	Genome	Goat	4
*Neocallimastix frontalis*	Hef5	GGXJ00000000	Transcriptome	Cow	This study
*Neocallimastix* sp.	G3	GGXC00000000	Transcriptome	Sheep	This study

## RESULTS

### Divergence time estimation and phylogenomic relationship of Neocallimastigomycota.

**FIG 1:**
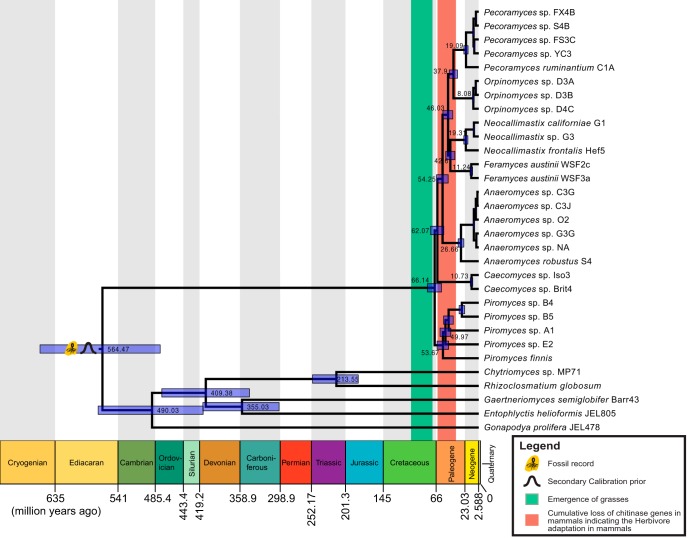
Bayesian phylogenomic maximum clade credibility tree of Neocallimastigomycota with divergence time estimation. All clades are fully supported by Bayesian posterior probabilities (BPP). For clarity, mean ages and 95% highest-probability density ranges (blue bars) are denoted on the nodes above the rank of genus.

Phylogenomic analysis placed the 26 AGF taxa into a single monophyletic clade with strong support of Bayesian posterior probability (1.0/1.0) and maximum likelihood bootstrap value (100%) (Fig. 1; see also Fig. S1 in the supplemental material). All AGF genera (Anaeromyces, Caecomyces, Feramyces, Neocallimastix, Orpinomyces, Pecoramyces, and Piromyces) included in this study formed individual monophyletic clades that were also supported by both Bayesian (Fig. 1) and maximum likelihood (Fig. S1) analyses. A conflict in the tree topology between the two phylogenetic reconstructions is the placement of the *Caecomyces* clade. This lineage is sister to the rest of the Neocallimastigomycota in the maximum likelihood tree (Fig. S1), while the *Caecomyces* position is swapped with *Piromyces* in the Bayesian phylogeny (Fig. 1). This is likely due to short internode distances, which suggests a rapid radiation of the ancestors of the two genera. The relative short bar of the highest-probability density (HPD) on the node of the AGF clade (Fig. 1) suggests the integrative natural history of this group of fungi and the outperforming resolving power of the genome-wide data in the molecular dating analyses.

10.1128/mSystems.00247-19.1 FIG S1 Maximum likelihood phylogenetic tree of Neocallimastigomycota using Chytridiomycota as the outgroup. All bootstrap values (out of 100) are labeled on the branches. Download FIG S1, PDF file, 0.2 MB. Copyright © 2019 Wang et al. 2019 Wang et al. This content is distributed under the terms of the Creative Commons Attribution 4.0 International license.

The divergence time of the Neocallimastigomycota clade is estimated at the Cretaceous/Paleogene (K/Pg) period boundary 66 (±10) million years ago (Mya) (Fig. 1). The chronogram (Fig. 1) displays a long branch leading to the emergence of the AGF clade, which extends from the end of Ediacaran period (∼564 Mya) to the K/Pg period boundary (∼66 Mya). This suggests that the extant members of AGF did not emerge until recently and then rapidly radiated into separate clades in the Paleogene. The estimated time frame for AGF divergence broadly coincides with the age of the grasses (70 to 95 Mya), previously estimated using molecular (nuclear and chloroplast) markers, and calibrated using fossils from pollen and dinosaur coprolite as well as the breakup time of the Gondwana (22–26). In addition, this inferred AGF divergence time also coincides with a major diet change of placental mammals, the transition from a primarily insectivorous to an herbivorous and omnivorous lifestyle. The loss of chitinase gene diversity, estimated to occurred from the K/Pg period boundary (66 Mya) to the mid-Paleogene period (34 Mya) (Fig. 1), is widely seen as a consequence of such a transition (27). Collectively, these overlapping estimates suggest that the evolution of the symbiotic association between herbivorous mammals and rumen fungi is tightly linked with the evolution of forage grasses and mammalian dietary transitions within a 66- to 95-Mya time frame. The exact chronology of these three divergence or transition events cannot be accurately determined partially due to the intervals of the estimates (Fig. 1). However, the dates inferred from phylogenetic analyses are consistent with the hypothesis that rumen fungi have played important roles in the dietary transition of some mammals to acquire nutrition from forage grasses.

### Genome-wide comparison of protein domains and homologous genes.

**FIG 2:**
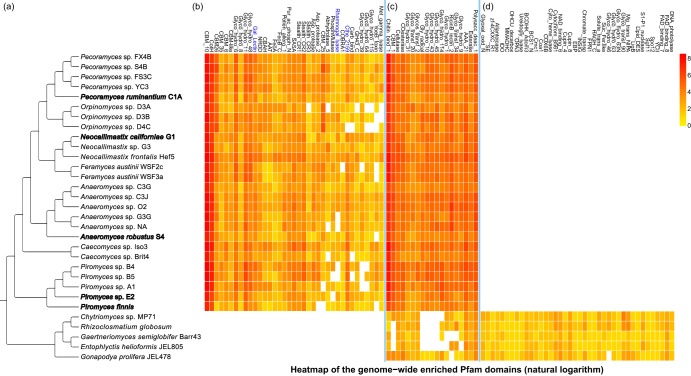
Cladogram and heatmap enrichment of the Pfam domains between Neocallimastigomycota and Chytridiomycota. (a) Cladogram showing the phylogenetic relationship of the compared taxa (Neocallimastigomycota genomes are in bold). (b) Heatmap plot of natural logarithm of the domain copy numbers showing the ones uniquely gained in Neocallimastigomycota (Pfam domains highlighted in this study are in blue). (c) Pfam domains highly enriched in Neocallimastigomycota. (d) Pfam domains absent in Neocallimastigomycota (presented domains are partial; see Table S1 for the full list).

Comparative genomic analysis between AGF and their non-rumen-associated chytrid relatives (Fig. 2) identified 40 Pfam domains that are unique to the AGF, representing 0.67% of the total number of Pfams (5,980) in the AGF pangenome-transcriptome (Table S1 and Fig. 2b). The predicted functions of these domains include anaerobic ribonucleotide reductase (NRDD), metal transport and binding (FeoA and FeoB_C), carbohydrate binding (e.g., CBM_10, CBM-like, and Cthe_2159), atypical protein kinase (CotH), and glycoside hydrolase (e.g., Glyco_hydro_6 and Glyco_hydro_11) (Table S1 and Fig. 2b). In addition to these 40 unique AGF domains, many additional Pfams were also enriched in the AGF. Such domains mediate polysaccharide degradation and monosaccharide fermentations (Fig. 2c), including Chitin_binding_1, CBM_1, Cellulase, Glyco_hydro_10, Gly_radical, RicinB_lectin_2, Esterase, and Polysacc_deac_1 domains. Further, our analysis also identified 106 Pfam domains that are not present in AGF genomes and transcriptomes but found in sister Chytridiomycota. Most of these missing domains are related to oxidation reactions on cytochromes and mitochondria; instead, they possess specialized organelles called hydrogenosomes conducting metabolism under anaerobic conditions (6) (Table S1 and Fig. 2d). In addition, domains involved in the biosynthesis of nicotinic acid, uric acid, and photolyase, in purine catabolism, and in pathways of ureidoglycolate and kynurenine are also found to be absent in AGF species. Similar patterns were also identified in the comparison of homologous genes (Fig. S2).

10.1128/mSystems.00247-19.2 FIG S2 Presence (dark gray) and absence (light gray) of the homologous gene families across the genomes (and transcriptomes) of Neocallimastigomycota and Chytridiomycota. The 4,824 gene families were selected as universal homologous genes that present at least 21 out of the 26 Neocallimastigomycota genomes (and transcriptomes) with missing no more than 1 of the 5 included Chytridiomycota genomes. In addition, it also includes the unique gene families that are strictly absence from all Chytridiomycota but encoded by the Neocallimastigomycota (missing no more than 5 out of the 26 taxa). Download FIG S2, PDF file, 2.5 MB. Copyright © 2019 Wang et al. 2019 Wang et al. This content is distributed under the terms of the Creative Commons Attribution 4.0 International license.

10.1128/mSystems.00247-19.8 TABLE S1 List of Pfam domain names with annotated functions for the ones uniquely maintained or lost in Neocallimastigomycota (AGF) compared to Chytridiomycota. Download Table S1, DOCX file, 0.1 MB. Copyright © 2019 Wang et al. 2019 Wang et al. This content is distributed under the terms of the Creative Commons Attribution 4.0 International license.

A permissive criterion, allowing some missing copies, found a total of 2,728 gene families shared between AGF and chytrids. We discovered that 1,709 additional gene families are shared among AGF genomes (each gene presents in at least 21 out of the total 26 taxa) but absent in other chytrids, while another 367 families are missing in AGF members but present in the other chytrid lineages.

### Genomic interactions within the rumen of mammalian herbivores.

We focused on three Pfam domains (Cthe_2159, Gal_Lectin, and Rhamnogal_lyase) that are unique to the Neocallimastigomycota and previously not observed in fungal genomes. Phylogenetic analyses support a horizontal transfer of Cthe_2159 from rumen bacteria into AGF, followed by potential gene fusion to deliver eukaryotic specific functions. Similarly, analysis of Gal_Lectin domain copies in AGF suggests they were acquired from animal donor lineages. A similarity search of the AGF Rhamnogal_lyase domain finds most similar copies in plant genomes, and phylogenetic analysis indicates that the AGF polysaccharide lyase domain is distinct and not orthologous to related enzymes in other fungi.

### A bacteria-like biomass-binding and putatively polysaccharide lyase domain, Cthe_2159.

**FIG 3:**
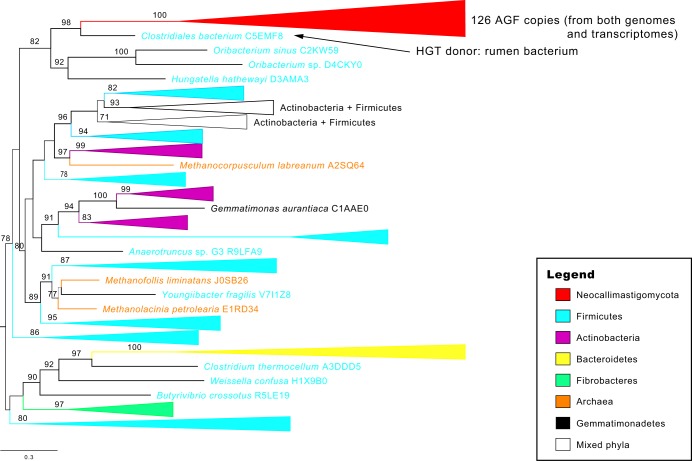
Midpoint-rooted phylogenetic tree of the Cthe_2159 domain. All 126 Neocallimastigomycota (AGF) copies (copies that have >90% identities have been removed) form a single clade (red) indicating the HGT donor, *Clostridiales bacterium* C5EMF8 (an obligate rumen bacterium), with strong support of maximum likelihood bootstrap (98/100). Included bacterial lineages were assigned different colors according to their phylogenetic classification (see legend for detailed information; the complete tree with all tip information is shown in Fig. S3).

**FIG 4:**
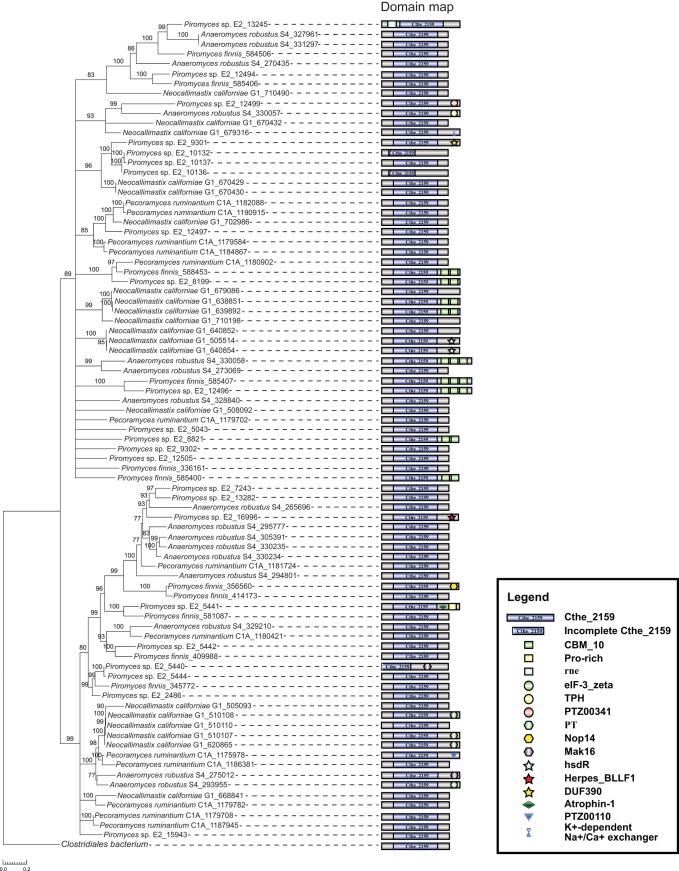
Phylogenetic tree of the 83 Cthe_2159 domains identified in five AGF genomes. Coding sequence identifiers used in the original annotation files are labeled with species names in tree tips. The tree is rooted with the closest related bacterial homolog found in *Clostridiales bacterium*. The domain map on the right shows the conserved domains produced by the Cthe_2159-containing genes.

The Cthe_2159 domain was originally characterized as a polysaccharide lyase-like protein in the thermophilic and biomass-degrading bacterium Clostridium thermocellum (28). Proteins of the Cthe_2159 domain are beta-helix proteins with the ability to bind celluloses and acid sugars (polygalacturonic acid, a major component of the pectin), and homologs are primarily found in archaeal and bacterial genomes. Notably, a total 583 copies of the Cthe_2159 domain were identified in 5 genomes and 21 transcriptomes of AGF taxa, but this was reduced to a set of 126 clusters based on overall protein similarity (>90%) due to redundancy in transcriptome assemblies. This domain is absent in all other eukaryotic genomes examined in this study (Fig. 3 and Table 2). A phylogenetic tree of Cthe_2159 homologs identified from archaea, bacteria, and AGF suggests that the AGF Cthe_2159 domains were acquired from bacteria through HGT (Fig. 3). The likely donor was a Gram-positive firmicute (*Clostridiales*) (maximum likelihood bootstrap value, 98%), and the closest protein copies of Cthe_2159 domains are encoded in the Oribacterium sinus, Oribacterium sp., and Hungatella hathewayi genomes (Fig. 3). Members of the order *Clostridiales* are integral members of the rumen microbiome. Four of these AGF Cthe_2159 domain-containing genes also encode eukaryotic Pfam protein domains (Atrophin-1, eIF-3_zeta, Nop14, and TPH) at the 3′ position of the Cthe_2159 domain. We hypothesize that these domains are the result of fusion after the acquisition of Cthe_2159 domain. The putative functions of these additional domains include initiation of the eukaryotic translation, maturation of 18S rRNA, production of 40S ribosome, and meiosis-specific activities (Fig. 4). Approximately 30% of these AGF Cthe_2159 gene models possess between 1 and 2 introns, but there is limited spliced transcript evidence to provide confidence in the gene structures, so the apparent intron gains could be artifacts of genome assembly or annotation (Text S1) (3, 4).

**TABLE 2:** Distribution of the three studied domains in the fungal kingdom

Phylum	No. of examined genomes	No. of domains		
		Cthe_2159	Gal_Lectin	Rhamnogal_lyase
Ascomycota	652	0	0	0
Basidiomycota	324	0	0	0
Mucoromycota	76	0	0	0
Zoopagomycota	23	0	0	0
Chytridiomycota	14	0	0	0
Neocallimastigomycota	5	95	67	26
Blastocladiomycota	4	0	0	0
Cryptomycota	1	0	0	0
Microsporidia	22	0	0	0
Total	1,121			

10.1128/mSystems.00247-19.10 TEXT S1 Supplemental results and methods for detecting putative intron gain in AGF Cthe_2159 genes. Download Text S1, DOCX file, 0.1 MB. Copyright © 2019 Wang et al. 2019 Wang et al. This content is distributed under the terms of the Creative Commons Attribution 4.0 International license.

10.1128/mSystems.00247-19.3 FIG S3 Midpoint-rooted phylogenetic tree of the Cthe_2159 domain encoded by the Neocallimastigomycota (red). All 126 Neocallimastigomycota (AGF) copies form a single clade (red) indicating the HGT donor, *Clostridiales bacterium* C5EMF8 (an obligate rumen bacterium), with strong support of maximum likelihood bootstrap (98/100). Download FIG S3, PDF file, 0.1 MB. Copyright © 2019 Wang et al. 2019 Wang et al. This content is distributed under the terms of the Creative Commons Attribution 4.0 International license.

### An animal-like galactose binding lectin domain, Gal_Lectin.

**FIG 5:**
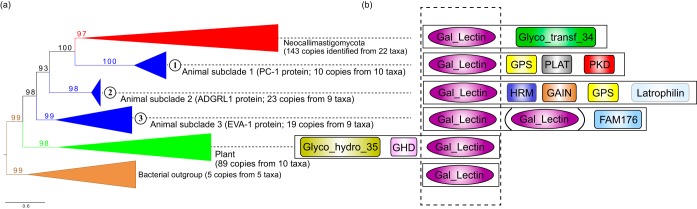
Phylogenetic tree of the animal-like Gal-Lectin domain identified in Neocallimastigomycota. (a) Collapsed phylogenetic tree based on protein sequences (rooted with the bacterial outgroup), including clades of Neocallimastigomycota (red), animals (blue; three clades are labeled 1 to 3), plants (green), and bacteria (brown) (a complete tree with all tip information is shown in Fig. S4). (b) Schematic diagrams showing the Gal_Lectin and other conserved domains on the same protein in each clade individually (dotted box highlights the aligned region used to produce the phylogenetic tree).

Gal_Lectin domains were found in AGF genomes universally and absent in all other examined chytrid and fungal genomes (Table 2). Phylogenetic analysis recovered a monophyletic AGF Gal_Lectin clade which was not placed as a sister clade to the animals as expected for a fungal gene. Instead, it was embedded within the animal homologs in the tree and allies with one subgroup, polycystin-1 (PC-1) (Fig. 5a). The three separate animal subclades contain protein members that harbor the Gal_Lectin domain but with dissimilar functions based on sequence homology (Fig. 5). The genomes of ruminant hosts (e.g., horse and sheep) of the AGF also contain three gene families with the Gal_Lectin domain, which can be observed in each of the animal subclades (Fig. 5). The proteins in the animal subclade 1 were annotated as PC-1 based on similarity to the human polycystic kidney disease (*PKD1*) genes. The members of the animal subclade 2 were searched by BLAST against the NCBI nonredundant protein database and identified as homologs of the adhesion G protein-coupled receptor L1/3 (ADGRL1). The animal subclade 3 contains homologs of the EVA-1 protein, most of which contain two adjacent copies of the Gal_Lectin domain. The three subgroups of animal Gal_Lectin domains are also flanked by disparate Pfam domains (Fig. 5b). The gene phylogeny suggests an animal PC-1 protein as the likely donor lineage for the AGF Gal_Lectin gene (Fig. 5a), based on its closest sister relationship. In addition, the AGF proteins also contain a Pfam Glyco_transf_34 domain (Fig. 5b) which is absent in all animal homologs of the Gal_Lectin-containing genes, suggesting its involvement in fungus-specific activities in the rumen.

10.1128/mSystems.00247-19.4 FIG S4 Phylogenetic tree of the animal-like Gal-Lectin domain identified in Neocallimastigomycota. Clades are colored as in Fig. 5 (Neocallimastigomycota in red, animals in blue, plants in green, and bacteria in brown). Download FIG S4, PDF file, 0.1 MB. Copyright © 2019 Wang et al. 2019 Wang et al. This content is distributed under the terms of the Creative Commons Attribution 4.0 International license.

10.1128/mSystems.00247-19.5 FIG S5 Phylogenetic tree of the AGF Gal-Lectin flanking domain Glyco_transf_34 (rooted with bacterium, in purple). AGF homologs are colored in red clustering with other fungal taxa (in black). Plant homologs are in green and protists in brown. Download FIG S5, PDF file, 0.1 MB. Copyright © 2019 Wang et al. 2019 Wang et al. This content is distributed under the terms of the Creative Commons Attribution 4.0 International license.

### A novel fungal rhamnogalacturonate lyase domain, Rhamnogal_lyase, in AGF.

**FIG 6:**
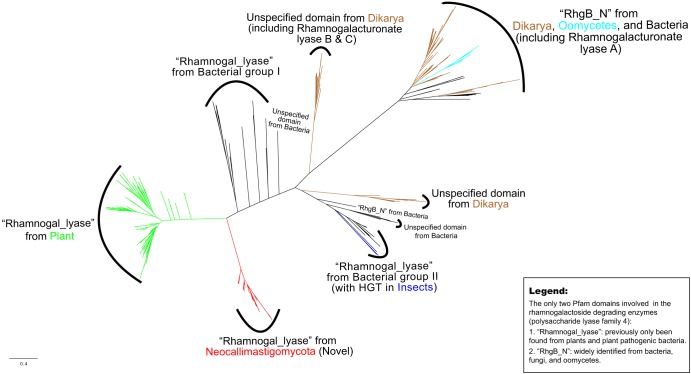
Radial phylogenetic tree of the Rhamnogal_lyase domain encoded by the Neocallimastigomycota (red). Plant copies are colored in green, and other homologous fungal genes are colored in brown. Oomycetes are in cyan, and animal copies only known in the mountain pine beetle *Dendroctonus ponderosae* are in blue. Bacterial branches are in black. The tree also included homologs of RhgB_N and Rhamnogalacturonan lyase A, B, and C. Domain names are suggested using NCBI’s conserved domain search tool (cutoff 1E^−5^) with unaligned FASTA sequences (refer to Fig. S6 for a tree with detailed information).

In plants, the rhamnogalacturonate lyases are involved in the fruit ripening-related process, cell wall modification, and lateral root and root hair formation (29, 30). The Pfam database classifies two types of domains for rhamnogalactoside-degrading activity, Rhamnogal_lyase and RhgB_N. They are both N-terminal catalytic domains associated with the rhamnogalacturonan lyase protein (polysaccharide lyase family 4 [PL4]) and flanked persistently by the group of fn3_3 and carbohydrate-binding module (CBM)-like domains, with the particular function of degrading the rhamnogalacturonan I (RG-I) backbone of pectin. The Rhamnogal_lyase domain is found in the genomes of plants and plant-pathogenic bacteria (e.g., Dickeya dadantii, formerly Erwinia chrysanthemi), whereas the RhgB_N domain has a wider distribution and can be found in bacteria, fungi, and oomycetes (31). Sequence similarity searches using the Rhamnogal_lyase domain against various protein sequence databases (e.g., EnsEMBL, MycoCosm, and Pfam) returned no homolog in any other fungi (except the AGF members), which indicates that this domain is unique to AGF, plants, and bacteria. On the other hand, the RhgB_N domain is widely shared by Dikarya fungi, oomycetes, and bacteria. Although the RhgB_N and Rhamnogal_lyase domains are distantly related according to sequence similarity (24% between the copies of the Aspergillus nidulans and Anaeromyces robustus), they presumably share an origin due to the fact that they both physically located on the N-terminal region of the PL4 proteins and they have resembling functions to degrade the pectin RG-I region. The phylogenetic tree shows that although AGF Rhamnogal_lyase domains are more closely related to the plant homologs than to the clades of fungi and oomycetes, these AGF rhamnogalacturonate lyases likely have evolved a specific function in fungi (Fig. 6). The presence of the Rhamnogal_lyase domain in the rumen-associated fungi suggests that the AGF may support an ability to soften, modify, and degrade the plant pectin within the anaerobic rumen in a related but different way from plants.

10.1128/mSystems.00247-19.6 FIG S6 Midpoint-rooted phylogenetic tree of the Rhamnogal_lyase domain encoded by the Neocallimastigomycota (red). Labels are consistent with Fig. 6. Download FIG S6, PDF file, 1.1 MB. Copyright © 2019 Wang et al. 2019 Wang et al. This content is distributed under the terms of the Creative Commons Attribution 4.0 International license.

## DISCUSSION

Microbial diversity of ruminants is a research hot spot for development of bioenergy tools (2, 32, 33). The AGF are an important but understudied component of the ruminant microbiome, and their obligate anaerobic and relatively large (50 to 200 Mb) and AT rich (78 to 84%) genomes challenged the initial generation of genomic resources for the clade. In this study, we produced the most phylogenetically broad transcriptome sampling of the Neocallimastigomycota fungi to date to support phylogenomic and comparative analyses. Our results contribute new insights into the natural history and dynamic evolution of these cryptic ruminant gut fungi. The reconstructed phylogenomic species tree resolved previously unanswered questions about the evolutionary relationships of the members of the AGF. In addition, we provide the first estimation of the divergence time of AGF taxa, 66 (±10) Mya (Fig. 1), which is in remarkable concordance with the divergence of the forage Poaceae grasses (70 to 95 Mya) and dietary shifts in mammalian lineages (34 to 66 Mya) from insectivore to herbivore and omnivore. Grass evolution enabled the herbivory transition, and this diet adaptation drove an increase in the developmental and morphological complexity of the digestive tract, compartmentalization, and the development of dedicated anaerobic fermentation chambers (e.g., rumen and cecum) in the herbivorous alimentary tract to improve biomass degradation efficiency (34). This transition to plant-based (or plant-exclusive) diets required additional partnership with microbes since mammals lack cellulolytic and hemicellulolytic enzymes necessary to liberate sugars for absorption (5). In addition, the genome content comparisons help illustrate and predict new biological roles AGF play in the mammalian herbivore guts. The long branch that leads to the emergence of the Neocallimastigomycota clade indicates the distinctiveness of the extant group of obligate symbiotic fungi in the mammalian herbivores and implies the existence of undiscovered although possibly extinct relatives of the Neocallimastigomycota and Chytridiomycota (Fig. 1). Future environmental and metagenome sequence exploration of anaerobic environment testing for presence of these types of fungi may provide new observations that support their existence.

Our analyses identified multiple instances of Pfam domain gains (*n* = 40) and losses (*n* = 106) within the Neocallimastigomycota clade (Fig. 2 and Table S1). As the mRNA collected for most of the fungi was from isolates grown on a single substrate (cellobiose), these observations are limited to the genes expressed under this condition. More comprehensive sampling of growth conditions across developmental stages and substrates is important to fully categorize gene gain and loss. We have taken a conservative approach that considers genes with gain or loss patterns shared across multiple RNA sequencing (RNA-seq) data sets and additionally confirmed by gene content in the five available AGF genomes. This approach identified three AGF lineage-specific protein domains which are absent from all other examined fungal genomes (Table 2). Phylogenetic analyses support the hypothesis that they were acquired via HGT or other noncanonical events. Phylogenetic analyses of Cthe_2159 and Gal_Lectin domains indicate that they were separately transferred from the rumen bacteria and animal hosts horizontally (Fig. 3 and 5). Prior studies of multiple fungal lineages suggested that lineage-specific genes may have come from lateral acquisition (35–37). The absence of homologs in the entire fungal clades (except Neocallimastigomycota in this case) is a strong signal for their potential foreign origins. The absence of any fungal homologs of Cthe_2159 outside the AGF prevented us from testing the alternative hypotheses of a fungal origin of the domain with an approximately unbiased (AU) topology constraint test (38). In analyzing Gal_Lectin, we compared the likelihoods of a constrained tree where all animal sequences were forced to be monophyletic to the topology of the unconstrained tree. The AU test did not find the constrained tree to be a statistically significantly worse fit, so the alternative hypothesis that the orphan Gal_Lectin in Neocallimastigomycota is a result of multiple independent loss events in all other fungal lineages could not be rejected by this analysis. Since the genetic distance between Neocallimastigomycota and sister chytrid relatives is quite large (Fig. 1), this multiple-loss scenario seems less likely than a single gain event. These identified domains highlight the diverse genomic tools utilized by AGF which we predict have improved their lignocellulolytic capacities (through Cthe_2159 and Rhamnogal_lyase) and recognition of molecules or other cells (through Gal_Lectin) within the rumen. The presence of four different eukaryotic Pfam domains fused with these bacterium-originated Cthe_2159 genes in AGF suggests that the genes are truly eukaryotic and present in the fungal genomes (Fig. 4) and not a contamination artifact. Studies of intron gains and losses in fungal lineages have suggested the ancestor was intron rich, an observation that is supported by intron-rich chytrid genomes (39–41). Although introns are present in several Cthe_2159 gene models in the Neocallimastigomycota genomes and flanked with duplicated coding sequences (Piromyces sp. strain E2), there is little support of spliced mRNA transcripts originating from these loci, preventing us from confidently declaring these as recent intron insertions (Text S1 and Fig. S7).

10.1128/mSystems.00247-19.7 FIG S7 Putative intron insertion events identified in the Cthe_2159 genes in the five AGF genomes. (a) Phylogenetic tree of the 83 AGF Cthe_2159 domains based on protein sequences (rooted with the closest related bacterial homolog). Both intron numbers and domain maps are shown and associated with the tree tips. Highlighted clades are provided with detailed comparative analyses for intron insertions (yellow clade is shown in the panel b; green clades are unfolded in the panel c). (b) Three highly identical Cthe_2159 domains (PirE2_1_10132, PirE2_1_10136, and PirE2_1_10137) with various intron numbers (0 to 2) located on the same scaffold (scaffold_146) of the *Piromyces* sp. E2 genome. Inserted introns can be either within or outside the Cthe_2159 domain. Pieces of duplicated regions are found in CDS2 and adjacently connected with the intron, thus likely associated with the intron insertion event in the PirE2_1_10137 gene. This is also suggested by multiple-sequence alignments (at both the protein and nucleotide levels) and self dot plot analyses. (c) Three pairs of Cthe_2159 domain are found identical and flanked on a pair of homologous scaffolds in *Piromyces finnis* (scaffold_6) and *Piromyces* sp. E2 (scaffold_46) genomes separately. Multiple intron gain and loss events can be identified by comparing each of the three pairs. A recent gene duplication event (PirE2_1_5444 is a recent duplicate of the PirE2_1_5440 according to the phylogenetic tree in panel a) followed by intron insertion (in PirE2_1_5444) is also found with redundant coding sequences within the Cthe_2159 domain. Similar to panel b, a regional duplication associated with intron insertion is suggested by both multiple-sequence alignment and self dot plot analyses. (d) Transcriptome mapping result of the *Piromyces finnis* showing the region of Pirfi3_345772 (both annotated introns were not supported by the transcriptome data). (e) Similar transcriptome mapping result for the region Pirfi3_414173 in *Piromyces finnis* (both annotated introns were not supported by the transcriptome data). Download FIG S7, PDF file, 1.6 MB. Copyright © 2019 Wang et al. 2019 Wang et al. This content is distributed under the terms of the Creative Commons Attribution 4.0 International license.

The Cthe_2159 protein family binds cellulosic and pectic substrates in the anaerobic and thermophilic bacterium Clostridium thermocellum (28). The crystal structure of the Cthe_2159 domain suggests that it is a polysaccharide lyase family with similarity to pectate lyases in the PL9 family. The Rhamnogal_lyase domains primarily function in the facilitation of cell wall modification in plants (29). The domain in phytopathogenic bacteria functions to disorganize plant tissues and support invasion (42). Although we cannot identify an unambiguous donor lineage of the AGF Rhamnogal_lyase domains (Fig. 6), their gain is a synapomorphy of the extant AGF taxa and may contribute to the ability of these fungi to access polysaccharides in plant cell walls. Both Cthe_2159 and Rhamnogal_lyase (PL4 family) domains have putative function in pectin binding or degradation activity, which we interpret as an indication of the importance of deconstruction of pectin in the lifestyle of AGF in the rumen (Table 2 and Fig. 2). Pectin is abundant in primary cell walls and the middle lamella in both dicotyledonous plants (making up 20 to 35% dry weight) and grasses (2 to 10%), serving as protection for plant cells from degrading enzymes produced by animals (43–46). The removal of pectin can increase the exposed surface area of a plant cell wall and improve the accessibility of degradation enzymes to other polysaccharides (cellulose and hemicellulose) masked by pectin (47). The Cthe_2159 and Rhamnogal_lyase proteins may contribute to the high efficiency of the AGF biomass degradability by uncoupling the pectin that glues cells together, increasing the exposed surface areas, and thus allowing diverse polysaccharide enzymes to work on plant cells simultaneously in the rumen. The fungi may benefit from these acquired domains in their capacity as primary degraders of ingested forage (48). Further investigation of their role in the multiple processes that AGF perform to weaken forage fibers and release polysaccharides is warranted (49, 50).

The Gal_Lectin domain bears the phylogenetic hallmark of being acquired from an animal donor. Animals use galactose-binding lectins to recognize foreign entities (51) and participate in antimicrobial defenses (52, 53). Our results suggest that the Gal_Lectin domains in AGF are homologous and closely related to animal PC-1 proteins (Fig. 5a), which are transmembrane proteins functioning in cell recognition (54, 55). *In vitro*, PC-1 shows binding ability to carbohydrate matrices and collagen types I, II, and IV (56). We postulate that the acquisition of the animal-like Gal_Lectin domain contributes to the AGF abilities of cell-cell recognition and interaction with other microbes in the rumen. The syntenic relationship of the coding genes shows that the AGF Gal_Lectin domains are flanked by the Glyco_transf_34 domain, which lacks homologs in any other animals (Fig. 5b and S5). The AGF-equipped Glyco_transf_34 belongs to the galactosyltransferase GMA12/MNN10 family and may help catalyze the transfer of the sugar moieties in cooperating with the adjacent Gal_Lectin domain. Our investigation found that HGT has contributed to the AGF genome evolution, with donors from both prokaryotes and eukaryotes. HGT may have helped these fungi to acquire new functions and to thrive in the anaerobic gut as a key member of the microbial community degrading plant materials in animal hosts.

Other than the arsenal of diverse enzyme profiles, the AGF have also been known to use rhizoids and holdfasts to physically aid the fungal body to penetrate into the plant material deeply, which is superior to other rumen microorganisms in terms of efficiency (5, 57). Our study provides evidence that the rumen fungi are able to and have actively acquired functional domains from the animal hosts and coexisting anaerobic bacteria in the rumen. These exotic genetic elements encoded in Neocallimastigomycota genomes may contribute to the unique traits of these fungi which are distinct from their free-living relatives. The long branch leading to the recent radiation of Neocallimastigomycota (Fig. 1) also suggests an evolutionary trajectory distinct from those of the sister Chytridiomycota lineages. Living as gut dwellers in the strict anaerobic gut environment for over 66 million years, AGF have undergone reductive evolution on the mitochondria and eventually transformed it to a new organelle, the hydrosome (3, 32). Their ecological roles of AGF in such an extreme environment also endow their exceptional ability for plant degradation. The AGF use both physical (deconstruction of lignocelluloses) and biological (depolymerization) mechanisms before the fermentation of plant polysaccharides. These steps require diverse enzymes capable of breaking chemical bonds in carbohydrates, including cellulases, hemicellulases, ligninases, and pectinases (58). In turn, the acquisition of these enzymatic processes has driven the synapomorphic and autapomorphic characteristics described in the AGF. Currently, few close relatives have been found, and none have been cultured which subtend from the long branch. Environmental DNA investigations of extreme and anaerobic environment that may be a suitable niche of those Neocallimastigomycota-like microbes may reveal potential relatives (59). For example, a recent metagenomic survey from coastal marine sediments suggests that some operational taxonomic units (OTUs) could be assigned to Neocallimastigomycota using a 28S rRNA marker (60). Sampling of deep-sea habitats and marine mammalian herbivores could provide future discoveries of biodiversity and evolutionary importance for understanding the evolutionary trajectory of the Neocallimastigomycota.

## MATERIALS AND METHODS

### RNA extraction, sequencing, and data set preparation.

In total, 21 strains of Neocallimastigomycota fungi were cultured from cow, sheep, horse, and goat feces and rumen fluid of fistulated cows in the Stillwater, OK, area (8) (Table 1). These strains were maintained under anaerobic conditions using the modified Hungate method, as described previously (61–64). Culture purity was ensured by serial dilution and incubation at 39°C for 24 to 48 h, followed by inoculation and a second round of isolation. Cellobiose was the sole carbon source of the fungal culture prepared for RNA extraction. The total volume of RNA was harvested at early stationary phase (48 to 60 h postinoculation) using the MasterPure yeast RNA purification kit (Epicentre, Madison, WI, USA) and processed for transcriptomics sequencing using the Illumina HiSeq 2500 platform and 2 × 150-bp paired-end library by Novogene (Beijing, China).

The RNA-seq data were assembled into *de novo* transcript assemblies using Trinity (v2.6.6) and used to predict ORFs using TransDecoder (v5.0.2) (65, 66). The generated proteomes and corresponding coding sequences were used as input to phylogenomic and comparative genomic analyses.

The five published Neocallimastigomycota genome sequences were obtained from the Joint Genome Institute (JGI) MycoCosm database (67, 68). These are the sequences for Anaeromyces robustus S4, Neocallimastix californiae G1, Pecoramyces ruminantium C1A (synonym Orpinomyces sp.), Piromyces finnis (v3.0), and Piromyces sp. E2 (3, 4). Five outgroup Chytridiomycota taxa with sequenced genomes were chosen. These are Chytriomyces sp. strain MP 71, Entophlyctis helioformis JEL805, Gaertneriomyces semiglobifer Barr 43, Gonapodya prolifera JEL478, and Rhizoclosmatium globosum JEL800 (69, 70).

### Phylogenomics and divergence time estimation.

A set of 434 highly conserved and generally single-copy protein-coding genes in fungi (https://doi.org/10.5281/zenodo.1251476) were developed through efforts of the 1000 Fungal Genomes Project and identified as single-copy genes in orthologous clusters provided in the Joint Genome Institute MycoCosm site (67, 71, 72). These markers were used for phylogenomic analyses in the PHYling pipeline (https://doi.org/10.5281/zenodo.1257001). Profile-Hidden-Markov models of these markers were searched in the chytrid predicted protein sequences using HMMER3 (v3.1b2). A total of 426 (out of 434) conserved orthologous markers were identified with hmmsearch (cutoff = 1E^−10^) in the 26 Neocallimastigomycota and 5 Chytridiomycota. The identified protein sequence homologs in each species, for each phylogenetic marker, were aligned with hmmalign to the marker profile-HMM. The protein alignments were also back translated into codon alignments guided by the protein alignment using the tool bp_mrtrans.pl (73). The protein and coding sequences of the markers were concatenated into a superalignment with 426 partitions defined by each gene marker. The 426 gene partitions were further collapsed into 33 partitions by PartitionFinder v2.1.1 with a greedy search to find partitions with consistent phylogenetic signals (74). Phylogenetic trees were constructed from this superalignment and partition scheme with two methods, the maximum likelihood method, implemented in IQ-TREE (v1.5.5), and Bayesian inference method, implemented in BEAST (v1.8.4) (75, 76). Configuration files for divergence time estimation analysis were coded in BEAUti v1.8.4 using the 33 partitions and two calibration priors, (i) a direct fossil record of Chytridiomycota from the Rhynie Chert (407 Mya) (77, 78), and (ii) the emergence time of Chytridiomycota (573 to 770 Mya as 95% HPD) from earlier studies (69, 79, 80). The Birth-Death incomplete sampling tree model was employed for interspecies relationship analyses (81). Unlinked strict clock models were used for each partition. The archive of input files and analysis scripts used to perform the phylogenetic analyses are available at Zenodo (https://doi.org/10.5281/zenodo.1447225). Three independent runs were performed separately for 50 million generations each with random starting seeds. Sufficient effective sample size (ESS) (>200) values were obtained after the default burn-in (10%) for the final sampled trees. The maximum clade credibility (MCC) tree was compiled using TreeAnnotator v1.8.4.

### Identification of AGF-specific genes and Pfam domains.

Orthologous genes across the 31 genomes or transcriptomes were identified using a comparative genomic pipeline that utilized all-versus-all BLASTp (cutoff = 1E^−5^) to obtain the similarity pairs, orthAgogue to identify putative orthologous relationships, and the Markov clustering algorithm (MCL, using the inflation value of 1.5) to generate disjoint clusters and deployed in an analysis pipeline (https://doi.org/10.5281/zenodo.1447225) (82–84). Comparisons of the shared gene content of the orthologous clusters were performed among the Chytridiomycota lineages using a permissive strategy of counting a gene family as shared if it is missing in up to 5 of the 26 Neocallimastigomycota taxa and 1 of the 5 chytrid genomes. In this scenario, genes absent in all chytrid genomes and maintained by more than 21 out of the 26 Neocallimastigomycota genomes/transcriptomes are defined as AGF unique genes; on the other hand, genes missing from all Neocallimastigomycota and present in at least 4 out of the 5 chytrid genomes are treated as AGF lost genes.

Protein domains were identified by searching the predicted proteomes from each genome assembly or transcriptome assembly against the Protein Family (Pfam) database (v31.0). The enrichment heatmap of the Pfam domains across the included taxa was produced using the aheatmap function in the R package NMF based on the total copy number (based on hmmscan results of searches against the Pfam database using a cutoff E-value of 1e−2) count in each assembly (85). Genes only present in the AGF genomes and missing from all of the included free-living chytrids relatives were identified.

To identify genes in AGF that are likely important for interactions with mammalian hosts and plant material breakdown, we further compared the five available AGF genomes to the genomes of their animal hosts (e.g., sheep, horse, elephant, and yak) (https://www.broadinstitute.org/elephant/elephant-genome-project and references 86–88), the diet plant (e.g., moss, rice, palm, maize, and sorghum) (89–98) (Table S2), and the 1,165 available fungal genomes from the ongoing 1000 Fungal Genomes Project (http://1000.fungalgenomes.org; https://mycocosm.jgi.doe.gov) (18, 19, 67, 68). Comparison of AGF genes to host or plant genomes was intended to test if any copies were likely donated from these lineages by searching for high-identity nucleotide matches. To prioritize AGF genes that may have been laterally acquired from these hosts, a Python script (13) and similarity search tool BLAT (99) were applied to filter out DNA elements in AGF with higher similarity to animal or plant homologs than any fungal ones, excluding the AGF themselves. Candidate genes for lateral transfer were ranked by the combination of the two strategies. The candidate genes with an assigned functional or biological process annotation were analyzed with priority using phylogenetic reconstruction to infer their potential origin.

10.1128/mSystems.00247-19.9 TABLE S2 Genome information of the animal hosts and diet plants used in the study to infer the genetic elements in Neocallimastigomycota that have a foreign origin. Download Table S2, DOCX file, 0.1 MB. Copyright © 2019 Wang et al. 2019 Wang et al. This content is distributed under the terms of the Creative Commons Attribution 4.0 International license.

### Identification of homologous sequences and potential origin of HGT candidate loci.

Three Pfam domains, Cthe_2159, Gal_Lectin, and Rhamnogal_lyase, were identified to be unique to the AGF genomes compared to the Chytridiomycota fungi or all other fungal members. To predict the donor lineages for these putative HGT events, we searched more broadly for homologues in genome databases of plant, metazoa, fungi, bacteria, and protists in EnsEMBL (v37) (100) via the Web-implemented HMMER tool (https://www.ebi.ac.uk/Tools/hmmer/) (cutoff = 1E^−3^). Additional fungal homologues were found by searching the Department of Energy (DOE) JGI’s MycoCosm database (67, 68). The profile Hidden Markov Model tool phmmer in the HMMer package (101) was used to search for similar sequences in the 1,165 fungal genomes using the query of edge-trimmed domain sequences from *A. robustus* (cutoff = 1E^−3^).

Members of the RhgB_N sequences were obtained from the Pfam database classified in the RhgB_N (PF09284) family (31), along with the N-terminal sequences of the rhamnogalacturonate lyase families A, B, and C from GenBank (102–104). A single data set of RhgB_N and Rhamnogal_lyase family members from animals, fungi, plants, and bacteria was constructed from these searches. Domain names were confirmed using NCBI’s conserved domain search tool (cutoff = 1E^−5^) with unaligned FASTA sequences (105). Similarly, homologs of the Gal_Lectin and Cthe_2159 domains were obtained by searching for similar sequences in the previously described genome databases and the categorized Pfam database (families of Gal_Lectin [PF02140] and Cthe_2159 [PF14262]). Homologous sequences containing the Cthe_2159 domain were only identified in archaea and bacteria, while the AGF copies are the first eukaryotic representatives identified with this domain. Homologs of the flanking domain Glyco_transf_34 were obtained similarly from EnsEMBL genome databases described above using the edge-trimmed domain sequence from *A. robustus* (cutoff = 1E^−5^). Highly similar sequences (>90%) were filtered using CD-HIT v4.6.4, followed by multiple-sequence alignment with MUSCLE v3.8.31 (106, 107).

### Phylogenetic analyses of the HGT candidates.

In total, 747 sequences of the rhamnogalacturanate degradation proteins (including both Rhamnogal_lyase and RhgB_N) were included in the alignment. For the other two domains, Gal_Lectin and Cthe_2159, the alignments include 297 and 234 unique variants, respectively. The Cthe_2159 domain-containing genes in the 5 AGF genomes were aligned separately using MUSCLE v3.8.31 in the Mesquite software (107, 108). Both the upstream and downstream flanking regions of the studied Pfam domain sequences were trimmed using the Mesquite software (108). Selection of the appropriate substitutional model, the maximum likelihood phylogenetic tree reconstruction, and the ultrafast bootstrapping (1,000 replicates) were conducted using the IQ-TREE v1.5.5 package (75, 109, 110).

### Data availability.

Assembled transcriptomes, raw Illumina read sequences, and isolate metadata are deposited in GenBank with the BioProject number PRJNA489922. All accession numbers are listed in Table 1. All generated RNA-seq reads were deposited in the Sequence Read Archive, and assembled transcriptomes were deposited in the Transcriptome Shotgun Assembly archive.
