# Supplementary figures and images for: Molecular Dating of the Emergence of Anaerobic Rumen Fungi and the Impact of Laterally Acquired Genes
Source: mSystems. 2019 Aug 27;4(4):e00247-19. doi: 10.1128/mSystems.00247-19 (PMC6712302; doi:10.1128/mSystems.00247-19)

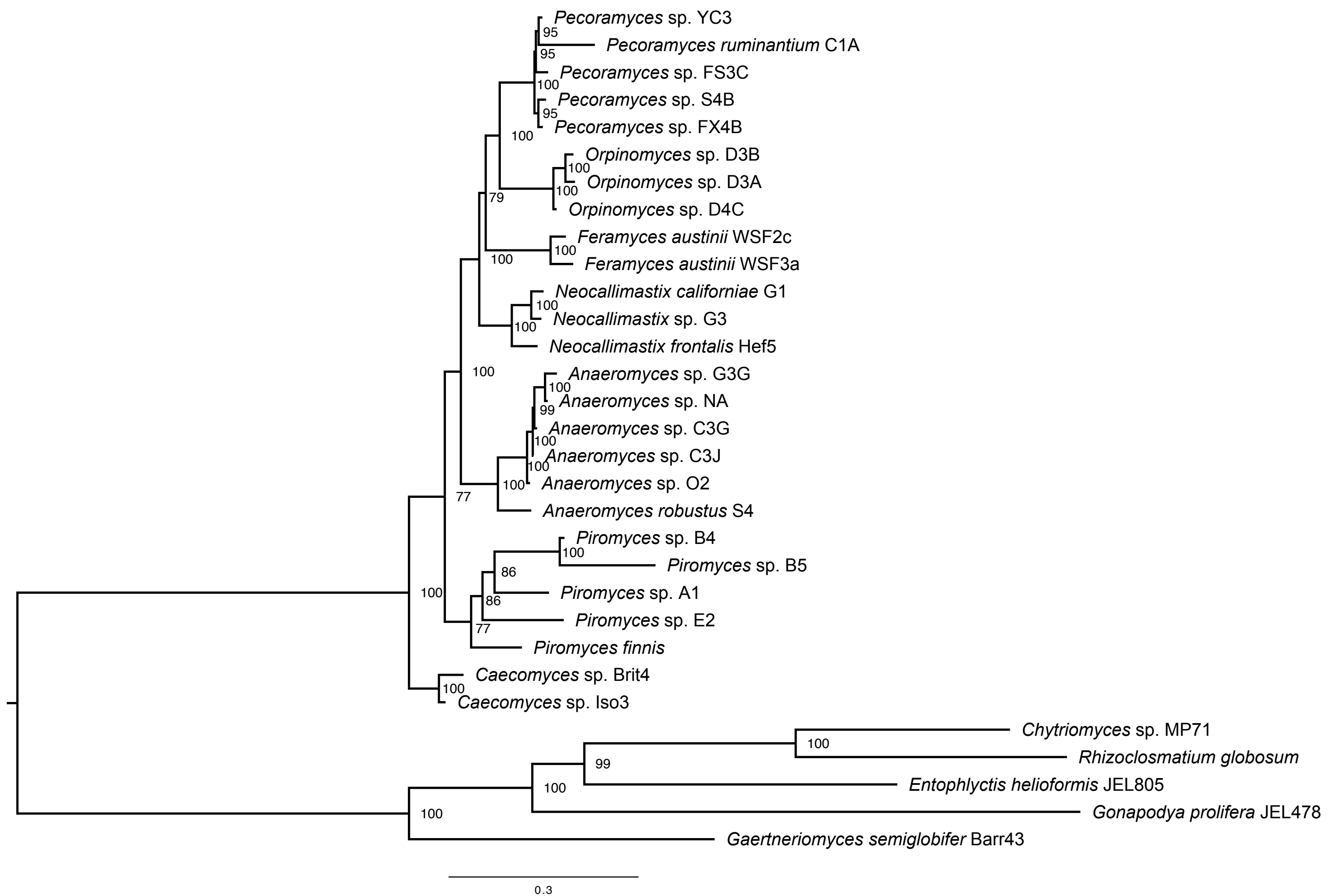

Supplement: FIG S1 [file mSystems.00247-19-sf001.pdf]

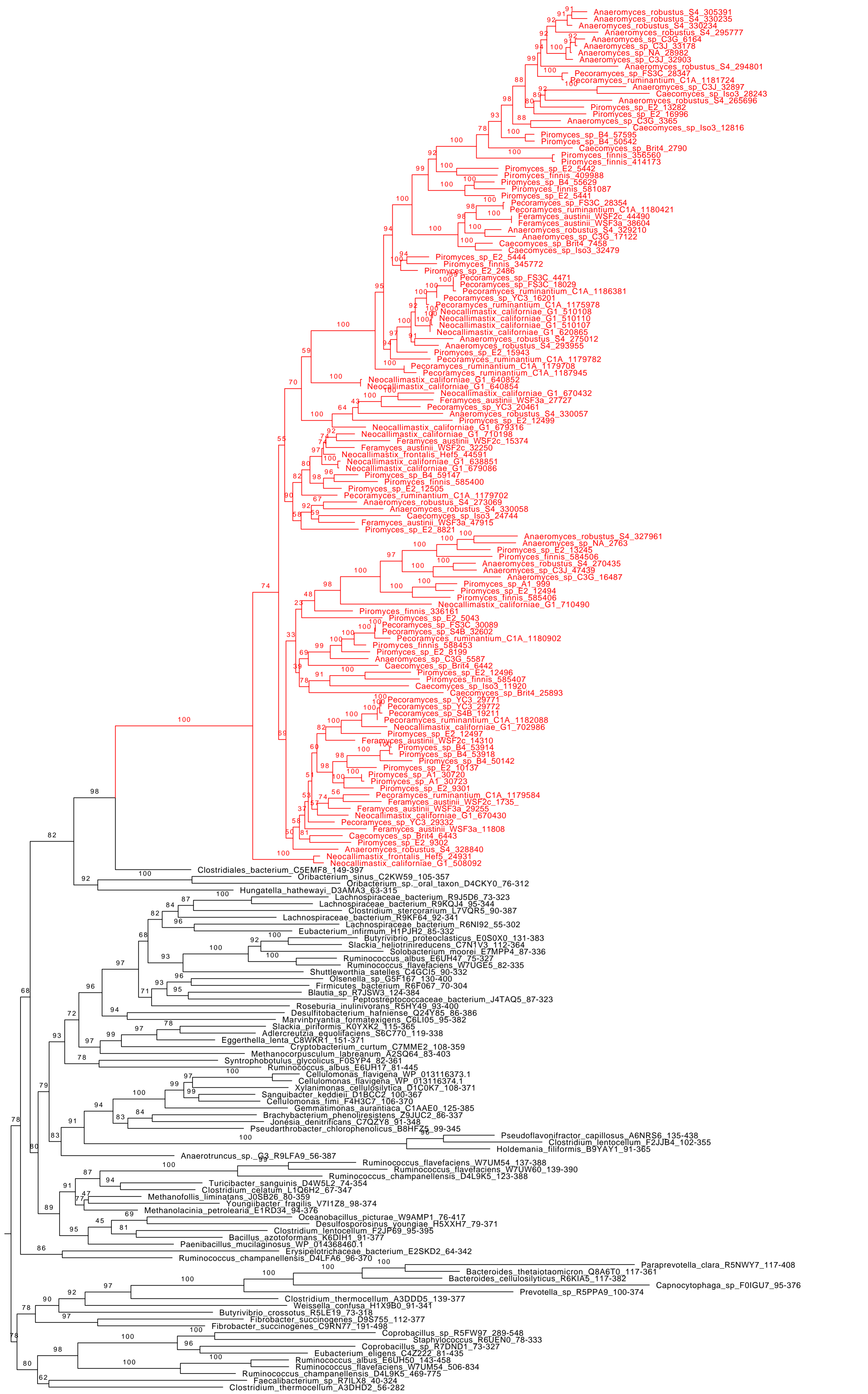

Supplement: FIG S3 [file mSystems.00247-19-sf003.pdf]

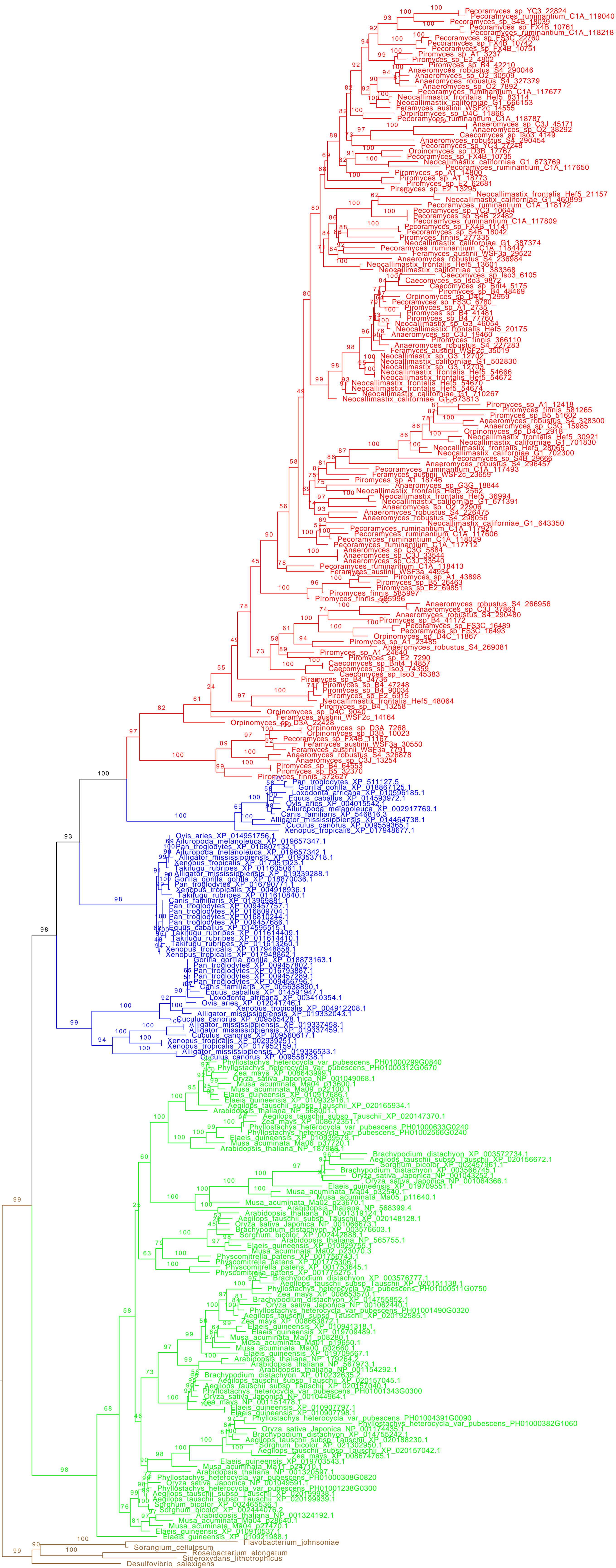

Supplement: FIG S4 [file mSystems.00247-19-sf004.pdf]

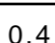

Supplement: FIG S5 [file mSystems.00247-19-sf005.pdf]

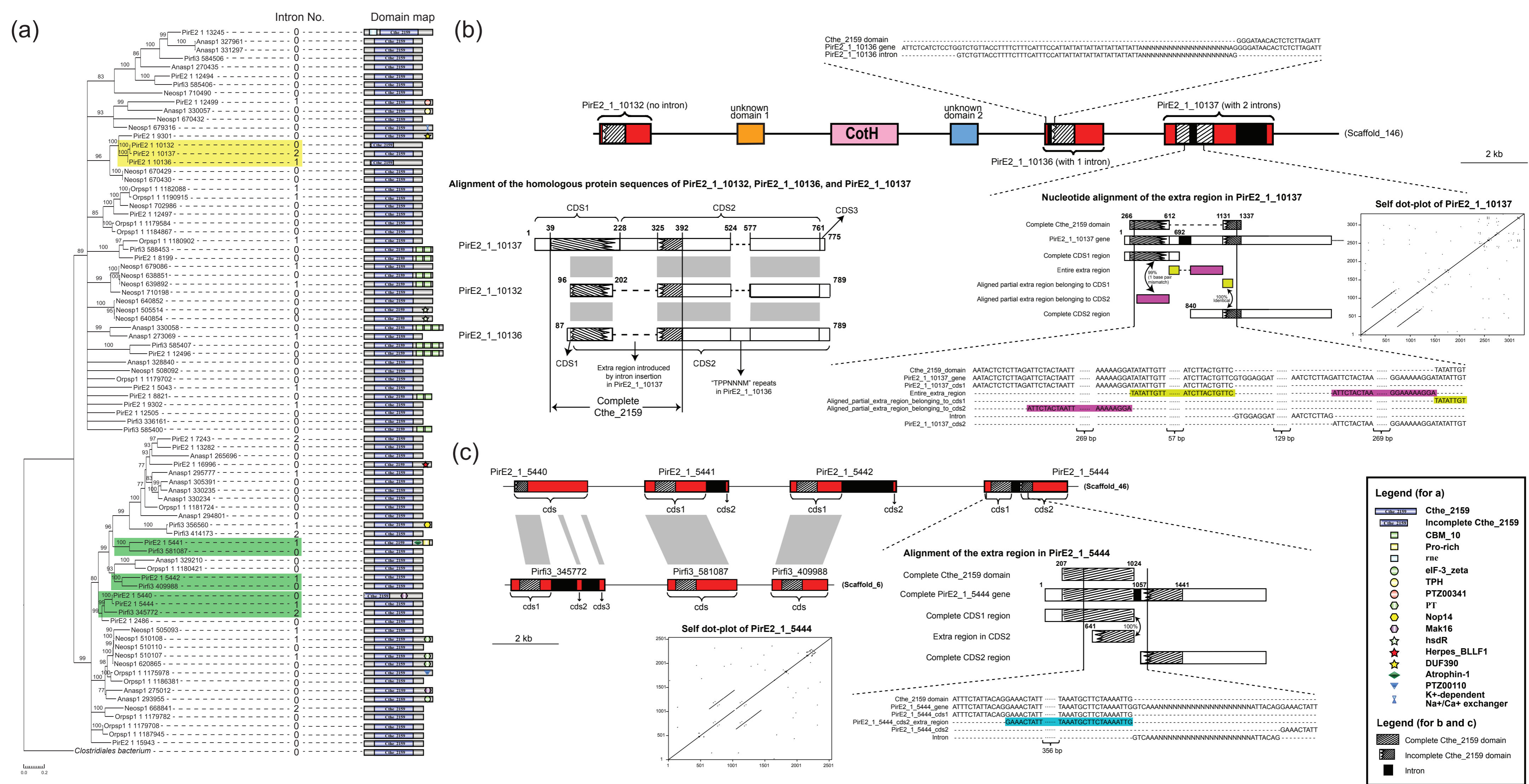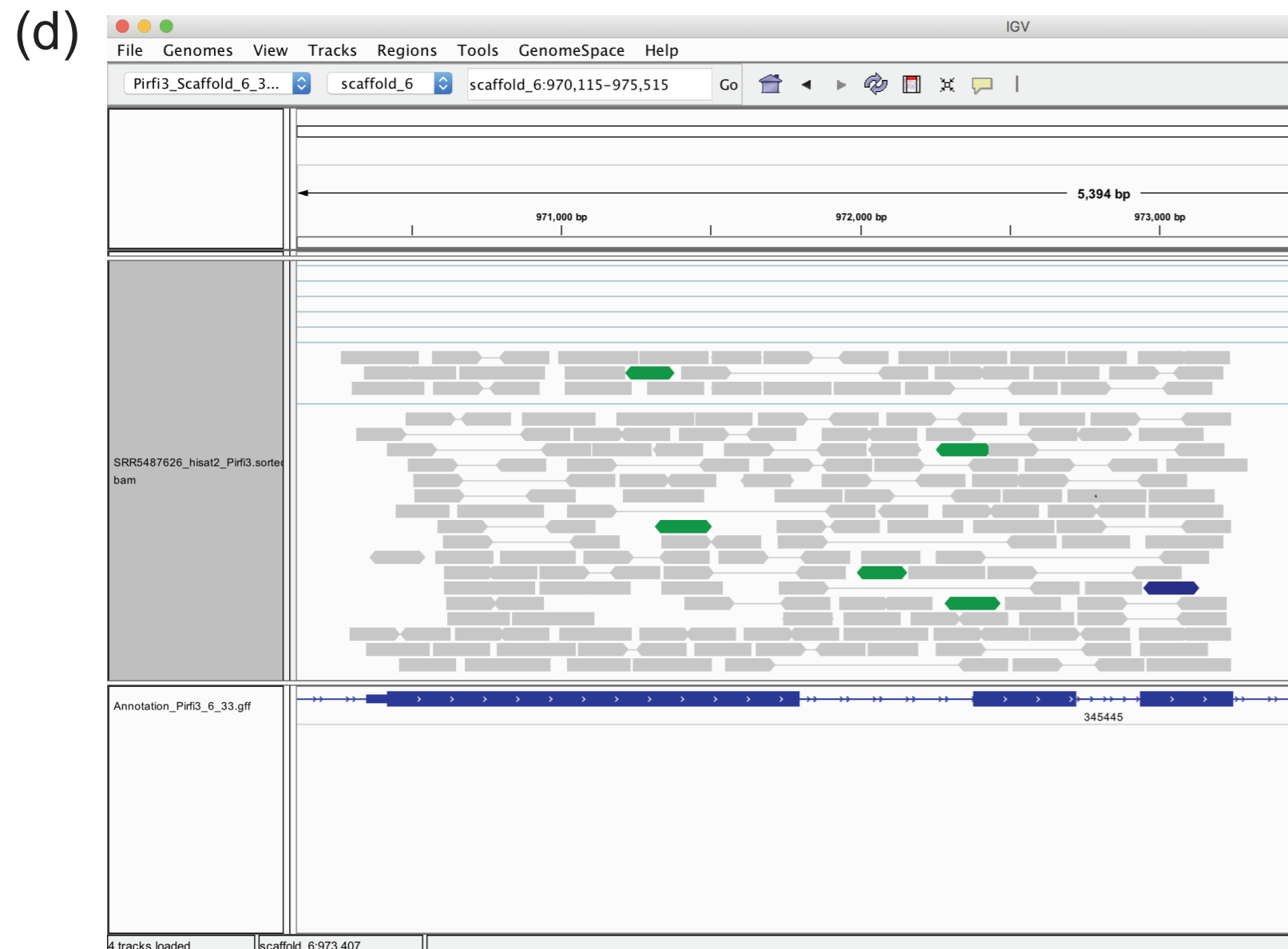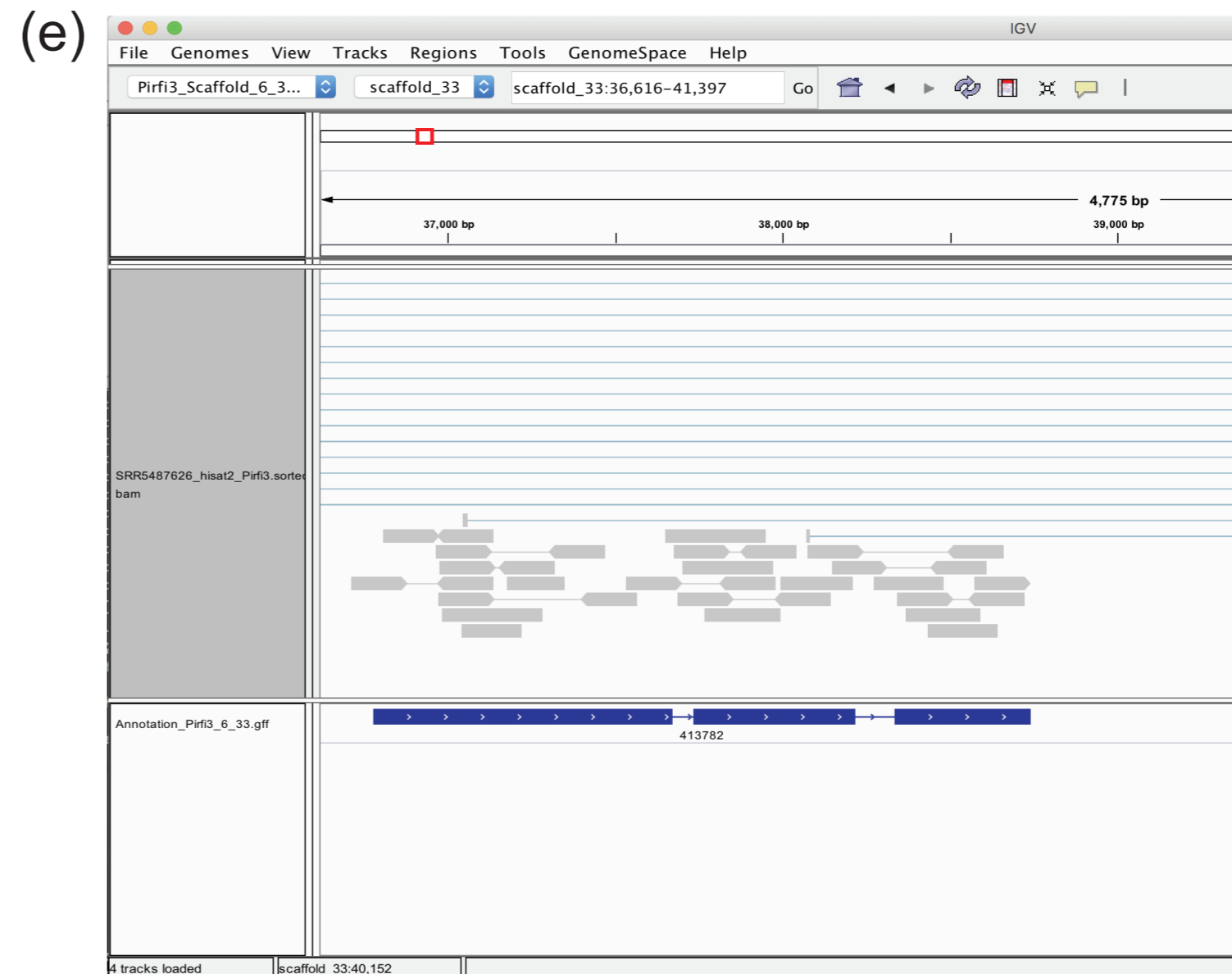

Supplement: FIG S7 [file mSystems.00247-19-sf007.pdf]
