# Supplementary material for: Molecular Dating of the Emergence of Anaerobic Rumen Fungi and the Impact of Laterally Acquired Genes
Source: mSystems. 2019 Aug 27;4(4):e00247-19. doi: 10.1128/mSystems.00247-19 (PMC6712302; doi:10.1128/mSystems.00247-19)
Supplement: FIG S2 [file mSystems.00247-19-sf002.pdf]

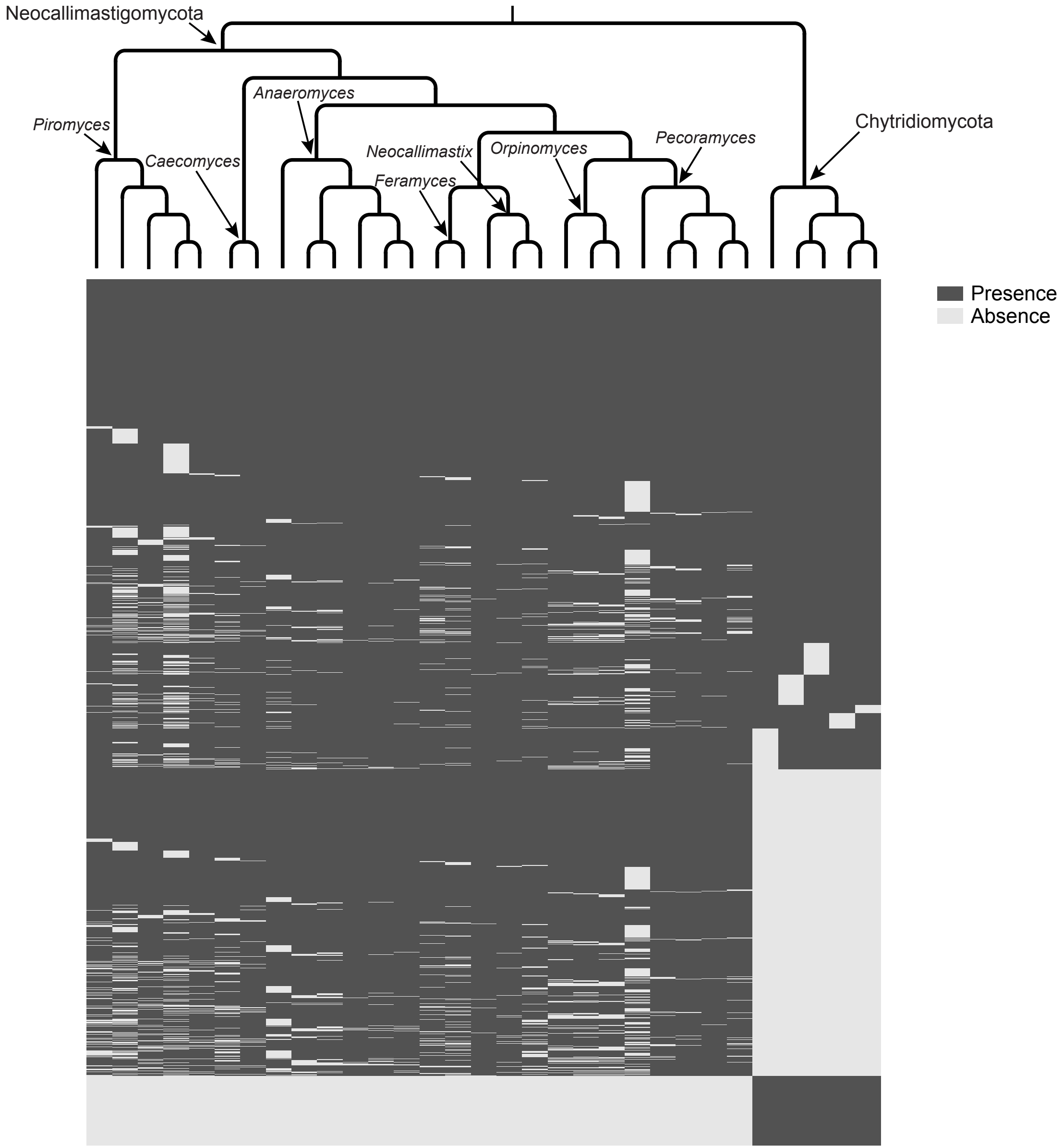

Neocallimastigomycota
Piromyces
Caecomyces
Anaeromyces
Neocallimastix
Feramyces
Orpinomyces
Pecoramyces
Chytridiomycota
Presence
Absence
