# Supplementary material for: Molecular Dating of the Emergence of Anaerobic Rumen Fungi and the Impact of Laterally Acquired Genes
Source: mSystems. 2019 Aug 27;4(4):e00247-19. doi: 10.1128/mSystems.00247-19 (PMC6712302; doi:10.1128/mSystems.00247-19)
Supplement: TABLE S1 [file mSystems.00247-19-st001.docx]

**Table S1.** List of Pfam domain names with annotated functions for the ones uniquely maintained or lost in Neocallimastigomycota (AGF) comparing to Chytridiomycota

| **AGF Unique Domains** | | **AGF Lost Domains** | | |
| --- | --- | --- | --- | --- |
| Domain | Function | Domain | Function | |
| AAT | Acyl-coenzyme A:6-aminopenicillanic acid acyl-transferase | 2OG-FeII_Oxy_3 | 2OG-Fe(II) oxygenase superfamily | |
| Abhydrolase_5 | Alpha/beta hydrolase superfamily, hydrolytic enzymes of widely differing phylogenetic origin and catalytic function that share a common fold. | 2OG-FeII_Oxy_4 | 2OG-Fe(II) oxygenase superfamily | |
| Asp_protease | Aspartic proteases are a catalytic type of protease enzymes that use an activated water molecule bound to one or more aspartate residues for catalysis of their peptide substrates. | 3-HAO | 3-hydroxyanthranilic acid dioxygenase, part of the kynurenine pathway for the degradation of tryptophan and the biosynthesis of nicotinic acid | |
| Asp_protease_2 | Aspartic proteases | ACAS_N | Acetyl-coenzyme A synthetase N-terminus | |
| Aspzincin_M35 | This is the catalytic region of aspzincins, a group of lysine-specific metallo-endopeptidases in the M35 family | Alg6_Alg8 | N-linked (asparagine-linked) glycosylation of proteins is mediated by a highly conserved pathway in eukaryotes, in which a lipid (dolichol phosphate)-linked oligosaccharide is assembled at the endoplasmic reticulum membrane prior to the transfer of the oligosaccharide moiety to the target asparagine residues. | |
| Carb_bind | This is a carbohydrate binding domain which has been shown in Schizosaccharomyces pombe to be required for septum localisation | Allantoicase | This enzyme belongs to the family of hydrolases, those acting on carbon-nitrogen bonds other than peptide bonds, specifically in linear amidines. The systematic name of this enzyme class is allantoate amidinohydrolase. This enzyme participates in purine metabolism by facilitating the utilization of purines as secondary nitrogen sources under nitrogen-limiting conditions. While purine degradation converges to uric acid in all vertebrates, its further degradation varies from species to species. Amphibians and microorganisms produce ammonia and carbon dioxide using the uricolytic pathway. Allantoicase performs the second step in this pathway catalyzing the conversion of allantoate into ureidoglycolate and urea. | |
| CBAH | Choloylglycine hydrolase family | ATP_sub_h | ATP synthase complex subunit h | |
| CBM_10 | Carbohydrate-binding module family 10 (CBM10) is found in two distinct sets of proteins with different functions. Those found in aerobic bacteria bind cellulose (or other carbohydrates); but in anaerobic fungi they are protein binding domains, referred to as dockerin domains. The dockerin domains are believed to be responsible for the assembly of a multiprotein cellulase/hemicellulase complex, similar to the cellulosome found in certain anaerobic bacteria. | ATP-synt_10 | mitochondrial ATPase complex subunit ATP1 | |
| CBM_25 | Carbohydrate-binding module family 25 (CBM25) binds alpha-glucooligosaccharides, particularly those containing alpha-1,6 linkages, and granular starch. | ATP11 | Atp11p is a molecular chaperone of the mitochondrial matrix that participates in the biogenesis pathway to form F1, the catalytic unit of the ATP synthase | |
| CBM_35 | This is a mannan-specific carbohydrate binding domain, previously known as the X4 module. Unlike other carbohydrate binding modules, binding to substrate causes a conformational change | B12D | NADH dehydrogenase (ubiquinone) | |
| CBM_6 | Carbohydrate-binding module family 6 (CBM6) is unusual in that is contains two substrate-binding sites, cleft A and cleft B. Cellvibrio mixtus endoglucanase 5A contains two CBM6 domains, the CBM6 domain at the C-terminus displays distinct ligand binding specificities in each of the sustrate-binding clefts. Both cleft A and cleft B can bind cello-oligosaccharides, laminarin preferentially binds in cleft A, xylooligosaccharides only bind in cleft A and beta1,4,-beta1,3-mixed linked glucans only bind in cleft B. | Band_7_C | C-terminal region of band_7 | |
| CBM-like | Polysaccharide lyase family 4, domain III | BCDHK_Adom3 | Mitochondrial branched-chain alpha-ketoacid dehydrogenase kinase | |
| CBM26 | CBM26 is a carbohydrate-binding module that binds starch | BCS1_N | Mitochondrial chaperone BCS1 | |
| CHB_HEX_C_1 | Chitobiase/beta-hexosaminidase C-terminal domain | Bot1p | Eukaryotic mitochondrial regulator protein | |
| CotH | CotH kinase protein, members of this family include the spore coat protein H (cotH). This protein is an atypical protein kinase that phosphorylates CotB and CotG | Chalcone_2 | Chalcone isomerase like | |
| Cthe_2159 | Carbohydrate-binding domain-containing protein | Chromate_transp | Members of this family probably act as chromate transporters. Members of this family are found in both bacteria and archaebacteria. The proteins are composed of one or two copies of this region. The alignment contains two conserved motifs, FGG and PGP. | |
| FeoA | This family includes FeoA a small protein, probably involved in Fe2+ transport [1]. This presumed short domain is also found at the C-terminus of a variety of metal dependent transcriptional regulators. This suggests that this domain may be metal-binding. In most cases this is likely to be either iron or manganese. | CIAPIN1 | Cytokine-induced anti-apoptosis inhibitor 1, Fe-S biogenesis. Anamorsin, subsequently named CIAPIN1 for cytokine-induced anti-apoptosis inhibitor 1, in humans is the homologue of yeast Dre2, a conserved soluble eukaryotic Fe-S cluster protein, that functions in cytosolic Fe-S protein biogenesis. It is found in both the cytoplasm and in the mitochondrial intermembrane space (IMS) | |
| FeoB_C | Ferrous iron transport protein B C terminus | Cmc1 | Cytochrome c oxidase biogenesis protein Cmc1 like | |
| FLYWCH | In molecular biology, the FLYWCH zinc finger is a zinc finger domain. It is found in a number of eukaryotic proteins. FLYWCH is a C2H2-type zinc finger characterised by five conserved hydrophobic residues, containing the conserved sequence motif: | Coa1 | Cytochrome oxidase complex assembly protein 1 | |
| Gal_Lectin | Galactose binding lectin domain | Complex1_LYR | NADH dehydrogenase (ubiquinone) | |
| Glyco_hydro_11 | Glycoside hydrolase family 11 CAZY GH_11 comprises enzymes with only one known activity, xylanase (EC 3.2.1.8). These enzymes were formerly known as cellulase family G | Complex1_LYR_2 | This is a family of proteins carrying the LYR motif of family Complex1_LYR, PF05347 likely to be involved in Fe-S cluster biogenesis in mitochondria. | |
| Glyco_hydro_48 | Glycoside hydrolase family 48 CAZY GH_48 comprises enzymes with several known activities; endoglucanase (EC 3.2.1.4); cellobiohydrolase (EC 3.2.1.91). | COQ7 | Ubiquinone biosynthesis protein COQ7 | |
| Glyco_hydro_6 | Glycoside hydrolase family 6 comprises enzymes with several known activities including endoglucanase (EC 3.2.1.4) and cellobiohydrolase (EC 3.2.1.91). These enzymes were formerly known as cellulase family B. The 3D structure of the enzymatic core of cellobiohydrolase II (CBHII) from the fungus Trichoderma reesei reveals an alpha-beta protein with a fold similar to the ubiquitous barrel topology first seen in triose phosphate isomerase. | COX15-CtaA | Cytochrome c oxidase | |
| Glyco_hydro_8 | Glycoside hydrolase family 8 CAZY GH_8 comprises enzymes with several known activities; endoglucanase (EC 3.2.1.4); lichenase (EC 3.2.1.73); chitosanase (EC 3.2.1.132). These enzymes were formerly known as cellulase family D. | COX6B | Cytochrome c oxidase, the last enzyme in the respiratory electron transport chain of mitochondria (or bacteria) located in the mitochondrial (or bacterial) membrane. | |
| GT-D | Glycosyltransferase GT-D fold | CtaG_Cox11 | Cytochrome c oxidase | |
| LRR_5 | A leucine-rich repeat (LRR) is a protein structural motif that forms an α/β horseshoe fold. It is composed of repeating 20–30 amino acid stretches that are unusually rich in the hydrophobic amino acid leucine. These repeats commonly fold together to form a solenoid protein domain, termed leucine-rich repeat domain. Typically, each repeat unit has beta strand-turn-alpha helix structure, and the assembled domain, composed of many such repeats, has a horseshoe shape with an interior parallel beta sheet and an exterior array of helices. One face of the beta sheet and one side of the helix array are exposed to solvent and are therefore dominated by hydrophilic residues. The region between the helices and sheets is the protein's hydrophobic core and is tightly sterically packed with leucine residues.  Leucine-rich repeats are frequently involved in the formation of protein–protein interactions. | Cupin_4 | Cupin superfamily protein | |
| NRDD | Anaerobic ribonucleoside-triphosphate reductase | Cupin_8 | This cupin like domain shares similarity to the JmjC domain, which catalyse a novel histone modification | |
| PDDEXK_2 | PD-(D/E)XK nuclease family transposase. These proteins are transposase proteins. | Cyto_heme_lyase | Holocytochrome-c synthase | |
| Pollen_allerg_1 | This family contains allergens lol PI, PII and PIII from Lolium perenne. | Cytochrom_B561 | Cytochrome b561 is an integral membrane protein responsible for electron transport, binding two heme groups non-covalently.[1] It is a family of ascorbate-dependent oxidoreductase enzymes | |
| Pur_ac_phosph_N | Purple acid Phosphatase, N-terminal domain | DAP3 | Mitochondrial ribosomal death-associated protein 3 | |
| Recombinase | This domain is usually found associated with PF00239 in putative integrases/recombinases of mobile genetic elements of diverse bacteria and phages. | DNA_photolyase | Deoxyribodipyrimidine photolyase (DNA photolyase) is a DNA repair enzyme. It binds to UV-damaged DNA containing pyrimidine dimers and, upon absorbing a near-UV photon (300 to 500 nm), breaks the cyclobutane ring joining the two pyrimidines of the dimer. | |
| Rhamnogal_lyase | Rhamnogalacturonate lyase ( EC:4.2.2.-) degrades the rhamnogalacturonan I (RG-I) backbone of pectin. This family contains mainly members from plants, but also contains the plant pathogen Erwinia chrysanthemi. | EBP | Emopamil binding protein, encodes a non-glycosylated type I integral membrane protein of endoplasmic reticulum and shows high level expression in epithelial tissues. The EBP protein has emopamil binding domains, including the sterol acceptor site and the catalytic centre, which show Delta8-Delta7 sterol isomerase activity. Human sterol isomerase, a homologue of mouse EBP, is suggested not only to play a role in cholesterol biosynthesis, but also to affect lipoprotein internalisation. | |
| Rubrerythrin | This domain has a ferritin-like fold | ETC_C1_NDUFA4 | NADH dehydrogenase (ubiquinone) | |
| rve | Integrase, Retroviral integrase (IN) is an enzyme produced by a retrovirus (such as HIV) that enables its genetic material to be integrated into the DNA of the infected cell. Retroviral INs are not to be confused with phage integrases, such as λ phage integrase (Int) (see site-specific recombination).  IN is a key component in the retroviral pre-integration complex (PIC). The complex of integrase bound to cognate viral DNA (vDNA) ends has been referred to as the intasome. | ETF_QO | Electron transfer flavoprotein-ubiquinone oxidoreductase, 4Fe-4S | |
| RVT_2 | Reverse transcriptase (RNA-dependent DNA polymerase), is usually indicative of a mobile element such as a retrotransposon or retrovirus. Reverse transcriptases occur in a variety of mobile elements, including retrotransposons, retroviruses, group II introns, bacterial msDNAs, hepadnaviruses, and caulimoviruses. This Pfam entry includes reverse transcriptases not recognised by the PF00078 model. | FAD_binding_7 | Photolyases (EC 4.1.99.3) are DNA repair enzymes that repair damage caused by exposure to ultraviolet light. | |
| SASA | Carbohydrate esterase, sialic acid-specific acetylesterase. Sialic acid acetylesterase in autoimmunity | FAD_binding_8 | FAD-binding domain | |
| Stealth_CR1 | Stealth protein CR1, Stealth_C1 is the first of several highly conserved regions on stealth proteins in metazoa and bacteria. There are up to four CR regions on all member proteins. CR1 carries a well-conserved IDVVYT sequence-motif. The domain is found in tandem with CR2, CR3 and CR4 on both potential metazoan hosts and pathogenic eubacterial species that are capsular polysaccharide phosphotransferases. The CR domains appear on eukaryotic proteins such as GNPTAB, N-acetylglucosamine-1-phosphotransferase subunits alpha/beta. Horizontal gene-transfer seems to have occurred between host and bacteria of these sequence-regions in order for the bacteria to evade detection by the host innate immune system | Ferric_reduct | Ferric reductase like transmembrane component. This family includes a common region in the transmembrane proteins mammalian cytochrome B-245 heavy chain (gp91-phox), ferric reductase transmembrane component in yeast and respiratory burst oxidase from mouse-ear cress. | |
| Stealth_CR2 | Stealth_CR2 is the second of several highly conserved regions on stealth proteins in metazoa and bacteria. There are up to four CR regions on all member proteins. CR2 carries a well-conserved NDD sequence-motif. The domain is found in tandem with CR1, CR3 and CR4 on both potential metazoan hosts and pathogenic eubacterial species that are capsular polysaccharide phosphotransferases. The CR domains appear on eukaryotic proteins such as GNPTAB, N-acetylglucosamine-1-phosphotransferase subunits alpha/beta. Horizontal gene-transfer seems to have occurred between host and bacteria of these sequence-regions in order for the bacteria to evade detection by the host innate immune system | FLILHELTA | protein of unknown function | |
| YoeB_toxin | A toxin-antitoxin system is a set of two or more closely linked genes that together encode both a protein 'poison' and a corresponding 'antidote'. When these systems are contained on plasmids – transferable genetic elements – they ensure that only the daughter cells that inherit the plasmid survive after cell division. If the plasmid is absent in a daughter cell, the unstable antitoxin is degraded and the stable toxic protein kills the new cell; this is known as 'post-segregational killing' (PSK). Toxin-antitoxin systems are widely distributed in prokaryotes, and organisms often have them in multiple copies. | FPN1 | Ferroportin1, that may play a role in iron export from the cell. This family may represent a number of transmembrane regions in Ferroportin1 | |
| ZinT | There is strong evidence for involvement of the ZinT domain in zinc homeostasis and management of zinc in the periplasm. It may also facilitate zinc uptake from the environment through interactions with the znuABC zinc transporter. It is regulated by the metalloregulator gene Zur (zinc uptake regulator).  The domain was originally discovered in the bacterial stress response to cadmium. Further studies have found that it binds to cadmium, zinc, nickel, and mercury, but not other common metals such as cobalt, copper, iron, and manganese. It may have a secondary function in managing heavy-metal toxicity. | Glyco_hydro_63 | Glycosyl hydrolase family 63 (CAZY GH_63) is a family of eukaryotic enzymes. They catalyse the specific cleavage of the non-reducing terminal glucose residue from Glc(3)Man(9)GlcNAc(2). Mannosyl oligosaccharide glucosidase EC 3.2.1.106 is the first enzyme in the N-linked oligosaccharide processing pathway. | |
|  |  | Glyco_hydro_63N | Glycosyl hydrolase family 63 (CAZY GH_63) is a family of eukaryotic enzymes. They catalyse the specific cleavage of the non-reducing terminal glucose residue from Glc(3)Man(9)GlcNAc(2). Mannosyl oligosaccharide glucosidase EC 3.2.1.106 is the first enzyme in the N-linked oligosaccharide processing pathway | |
|  |  | Glyco_transf_90 | This family of glycosyl transferases are specifically (mannosyl) glucuronoxylomannan/galactoxylomannan -beta 1,2-xylosyltransferases, EC:2.4.2.-. | |
|  |  | Glyoxal_oxid_N | Glyoxal oxidase N-terminus | |
|  |  | HAGH_C | Hydroxyacylglutathione hydrolase C-terminus. Substrate binding occurs at the interface between this domain and the catalytic domain | |
|  |  | IDO | Indoleamine 2,3-dioxygenase. Indoleamine 2,3-dioxygenase is the first and rate-limiting enzyme of tryptophan catabolism through the kynurenine pathway, thus causing depletion of tryptophan which can cause halted growth of microbes as well as T cells. | |
|  |  | IGR | IGR protein motif | |
|  |  | INSIG | Insulin-induced protein, found in the endoplasmic reticulum and bind the sterol-sensing domain of SREBP cleavage-activating protein (SCAP), preventing it from escorting SREBPs to the Golgi. Their combined action permits feedback regulation of cholesterol synthesis over a wide range of sterol concentrations. | |
|  |  | LIAS_N | LIAS_N is found as the N-terminal domain of the Radical_SAM family in the members that are lipoyl synthase enzymes, particularly the mitochondrial ones in metazoa but also those in bacteria. | |
|  |  | LigB | Catalytic LigB subunit of aromatic ring-opening dioxygenase | |
|  |  | Lipid_DES | Sphingolipid Delta4-desaturase (DES). Sphingolipids are important membrane signalling molecules involved in many different cellular functions in eukaryotes. | |
|  |  | MAM33 | Mitochondrial glycoprotein | |
|  |  | MCD | This family consists of several eukaryotic malonyl-CoA decarboxylase (MLYCD) proteins. Malonyl-CoA, in addition to being an intermediate in the de novo synthesis of fatty acids, is an inhibitor of carnitine palmitoyltransferase I, the enzyme that regulates the transfer of long-chain fatty acyl-CoA into mitochondria, where they are oxidised. | |
|  |  | Mg_trans_NIPA | Magnesium transporter NIPA | |
|  |  | Mgm101p | Mitochondrial genome maintenance MGM11 | |
|  |  | Mit_KHE1 | Mitochondrial K+-H+ exchange-related | |
|  |  | Mitofilin | Mitochondrial inner membrane protein | |
|  |  | MMADHC | Methylmalonic aciduria and homocystinuria type D protein | |
|  |  | Mo-co_dimer | Mo-co oxidoreductase dimerisation domain. This domain is found in molybdopterin cofactor (Mo-co) oxidoreductases. It is involved in dimer formation, and has an Ig-fold structure | |
|  |  | MOSC_N | MOSC N-terminal beta barrel domain, predicted sulfur-carrier domain | |
|  |  | MRP_L53 | 39S ribosomal protein L53 | |
|  |  | MRP-L20 | Mitochondrial ribosomal protein subunit L2 | |
|  |  | MRP-L28 | Mitochondrial ribosomal protein L28 | |
|  |  | MRP-L46 | 39S mitochondrial ribosomal protein L46 | |
|  |  | MRP-L47 | 39S mitochondrial ribosomal protein L47 | |
|  |  | MRP-S25 | Mitochondrial ribosomal protein S25 | |
|  |  | MTP18 | Mitochondrial 18 KDa protein | |
|  |  | MWFE | NADH dehydrogenase (ubiquinone), is an enzyme of the respiratory chains of myriad organisms from bacteria to humans that falls under the H+ or Na+-translocating NADH Dehydrogenase (NDH) Family (TC# 3.D.1), a member of the Na+ transporting Mrp superfamily. | |
|  |  | NAD_binding_6 | Ferric reductase NAD binding domain Provide feedback | |
|  |  | NADH-u_ox-rdase | NADH-ubiquinone oxidoreductase complex I, 21 kDa subunit | |
|  |  | NDUF_B7 | NADH dehydrogenase (ubiquinone) | |
|  |  | NTPase_I-T | protein of unknown function | |
|  |  | Ofd1_CTDD | Oxoglutarate and iron-dependent oxygenase degradation C-term | |
|  |  | OHCU_decarbox | In molecular biology 2-oxo-4-hydroxy-4-carboxy-5-ureidoimidazoline decarboxylase (OHCU decarboxylase) EC 4.1.1.n1 is an enzyme involved in purine catabolism | |
|  |  | OPA3 | Optic atrophy 3 protein, deficiency of which causes type III 3-methylglutaconic aciduria (MGA) in humans. This disease manifests with early bilateral optic atrophy, spasticity, extrapyramidal dysfunction, ataxia, and cognitive deficits, but normal longevity | |
|  |  | Pam17 | Mitochondrial import protein Pam17 | |
|  |  | PDZ_1 | These domains play a key role in the formation and function of signal transduction complexes. | |
|  |  | Peptidase_M76 | This is a family of metalloproteases. Proteins in this family are also annotated as Ku70-binding proteins. | |
|  |  | PET117 | PET assembly of cytochrome c oxidase | |
|  |  | Pet127 | Mitochondrial protein Pet127 | |
|  |  | Pet191_N | Cytochrome c oxidase | |
|  |  | Pirin_C | Pirin C-terminal cupin domain | |
|  |  | QCR10 | Ubiquinol-cytochrome-c reductase complex subunit | |
|  |  | Rad52_Rad22 | The DNA single-strand annealing proteins (SSAPs), such as RecT, Red-beta, ERF and Rad52, function in RecA-dependent and RecA-independent DNA recombination pathways | |
|  |  | RdRP | RNA dependent RNA polymerase, This family of proteins are eukaryotic RNA dependent RNA polymerases. These proteins are involved in post transcriptional gene silencing where they are thought to amplify dsRNA templates. | |
|  |  | Rib_5-P_isom_A | Ribose 5-phosphate isomerase A (phosphoriboisomerase A) | |
|  |  | Ribosomal_L33 | unknown | |
|  |  | Ribosomal_L34 | unknown | |
|  |  | Ribosomal_L36 | unknown | |
|  |  | RPOL_N | DNA-directed RNA polymerase N-terminal. This domain has a role in interaction with regions of upstream promoter DNA and the nascent RNA chain, leading to the processivity of the enzyme. This domain undergoes a structural change in the transition from initiation to elongation phase. The structural change results in abolition of the promoter binding site, creation of a channel accommodating the heteroduplex in the active site and formation of an exit tunnel which the RNA transcript passes through after peeling off the heteroduplex. | |
|  |  | S1-P1_nuclease | S1 P1 nuclease protein domain, which cleave RNA and single stranded DNA with no sequence specificity. They are found in both prokaryotes and eukaryotes and are thought to be associated in programmed cell death and also in tissue differentiation. Furthermore, they are secreted extracellular, that is, outside of the cell. Their function and distinguishing features mean they have potential in being exploited in the field of biotechnology. | |
|  |  | SCO1-SenC | This family is involved in biogenesis of respiratory and photosynthetic systems. | |
|  |  | SE | Squalene epoxidase. This domain is found in squalene epoxidase (SE) and related proteins which are found in taxonomically diverse groups of eukaryotes and also in bacteria. SE was first cloned from Saccharomyces cerevisiae where it was named ERG1. It contains a putative FAD binding site and is a key enzyme in the sterol biosynthetic pathway [1]. Putative transmembrane regions are found to the protein's C-terminus. | |
|  |  | Sgf11 | The Sgf11 family is a SAGA complex subunit in Saccharomyces cerevisiae. The SAGA complex is a multisubunit protein complex involved in transcriptional regulation. SAGA combines proteins involved in interactions with DNA-bound activators and TATA-binding protein (TBP), as well as enzymes for histone acetylation and deubiquitylation | |
|  |  | She9_MDM33 | mitochondrial inner membrane proteins with a role in inner mitochondrial membrane organisation and biogenesis | |
|  |  | Solute_trans_a | Organic solute transporter Ostalpha. This family is a transmembrane organic solute transport protein. In vertebrates these proteins form a complex with Ostbeta, and function as bile transporters [1]. In plants they may transport brassinosteroid-like compounds and act as regulators of cell death | |
|  |  | Spo12 | The Spo12 protein plays a regulatory role in two of the most fundamental processes of biology, mitosis and meiosis, and yet its biochemical function remains elusive. Spo12 is a nuclear protein. Spo12 is a component of the FEAR (Cdc fourteen early anaphase release) regulatory network, that promotes Cdc14 release from the nucleolus during early anaphase. The FEAR network is comprised of the polo kinase Cdc5, the separase Esp1, the kinetochore-associated protein Slk19, and Spo12. | |
|  |  | SPO22 | SPO22/ZIP4 in yeast is a meiosis specific protein involved in sporulation. It has been shown to regulate crossover distribution by promoting synaptonemal complex formation | |
|  |  | Suc_Fer-like | Sucrase/ferredoxin-like. This family contains a number of bacterial and eukaryotic proteins approximately 400 residues long that resemble ferredoxin and appear to have sucrolytic activity | |
|  |  | TIM21 | TIM21 interacts with the outer mitochondrial TOM complex and promotes the insertion of proteins into the inner mitochondrial membrane | |
|  |  | TMEM223 | Transmembrane protein 223 | |
|  |  | UcrQ | Coenzyme Q – cytochrome c reductase | |
|  |  | Ureidogly_lyase | Ureidoglycolate lyase, one of the enzymes that acts upon ureidoglycolate, an intermediate of purine catabolism, releasing urea. | |
|  |  | VRR_NUC | unknown | |
|  |  | YCII | YCII-related domain. This domain is suggested to play a role in transcription initiation (Bateman A per. obs.). This domain is named after the most conserved motif in the alignment. | |
|  |  | zf-4CXXC_R1 | Zinc-finger domain of monoamine-oxidase A repressor R1 | |
|  |  | zf-CHCC | NADH dehydrogenase (ubiquinone) | |
|  | | | | |
