# Supplementary material for: Molecular Dating of the Emergence of Anaerobic Rumen Fungi and the Impact of Laterally Acquired Genes
Source: mSystems. 2019 Aug 27;4(4):e00247-19. doi: 10.1128/mSystems.00247-19 (PMC6712302; doi:10.1128/mSystems.00247-19)
Supplement: TEXT S1 [file mSystems.00247-19-s0001.docx]

**Supplementary results for detecting putative intron gain in AGF “Cthe_2159” genes**

Approximately 30% of the “Cthe_2159” genes across all publicly available AGF genomes possess 1-2 introns according to the genome annotation (C. H. Haitjema et al., *Nat Microbiol* 2:1-8, 2017; N. H. Youssef et al., *Appl Environ Microbiol* 79:4620-4634, 2013). Phylogenetic analysis demonstrated that “Cthe_2159” genes locating on the same scaffold tend to cluster with each other with greater than 99/100 bootstrap supports, regardless of the intron numbers (0-2) they possess (Figure S7a). This suggests that the pattern of intron presence in AGF “Cthe_2159” is random without a major constraint evolutionary event above genus level. Some introns, especially in *Piromyces* sp. E2 are adjacent to duplicated fragments of coding sequences within the conserved “Cthe_2159” domain (e.g., PirE2_1_10137 in Figure S7b and PirE2_1_5444 in Figure S7c). The duplication is apparent by comparing alignments of the nucleotide sequences (Figure S7b-c). We hypothesize these introns represent a gain as a result of a local duplication event according to the proposed “Tandem Genomic Duplication” model of intron gains (P. Yenerall and L. Zhou, *Biol Direct* 7:1, 2012). Unfortunately, *Piromyces* sp. E2 strain lacks of transcriptome data, which could be used to confirm the reality of reported introns. Although Expressed Sequence Tag (EST) reads are available at JGI website, they are not sufficient to recover the interested regions (PirE2_1_10137 and PirE2_1_5444). In addition, the *Piromyces* sp. E2 genome is a pioneer assembly across the entire lineage (assembled in March 2011) using Sanger gDNA and shredded Velvet contigs (I. V. Grigoriev et al, *Nucleic Acids Res* 42:D699-D704, 2014; C. H. Haitjema et al., *Nat Microbiol* 2:1-8, 2017). We cannot exclude the possibility that the annotated introns and flanked duplications were due to assembly artifact, which pends future investigation using better quality genome assemblies if the strain is still alive and accessible.

The genome of *Piromyces finnis* represents the best quality of all AGF genomes with multiple transcriptomic characterization studies (C. H. Haitjema et al., *Nat Microbiol* 2:1-8, 2017; K. V. Solomon et al., *Science* 351:1192-1195, 2016). To seek evidence to prove the reality of annotated introns in “Cthe_2159” genes, we used splicing-site sensitive tool (Hisat2) to map the transcriptome reads to the *Piromyces finnis* genome assembly. At the interested regions where introns were reported in *Piromyces finnis*, we did not find supportive splicing evidence by examining the paired reads using Integrative Genomics Viewer (IGV) manually (Figure S7d-e) (J. T. Robinson et al., *Nat Biotechnol* 29:24-26, 2011). Although the negative evidence found in *Piromyces finnis* cannot be used to explain a crossed strain (*Piromyces* sp. E2) directly, especially considering that the reported introns in *Piromyces* sp. E2 are flanked by duplicated coding sequences, which is usually a strong sign for the intron insertion (P. Yenerall and L. Zhou, *Biol Direct* 7:1, 2012), the inconsistency between gene annotation and transcriptome evidence in *Piromyces finnis* alert us the intron presence in other AGF genomes may due to similar artifacts.

**Supplementary methods**

Introns in AGF “Cthe_2159” genes were identified by examining the genome annotation files (C. H. Haitjema et al., *Nat Microbiol* 2:1-8, 2017; N. H. Youssef et al., *Appl Environ Microbiol* 79:4620-4634, 2013). The “Cthe_2159” domain containing genes in the five AGF genomes were aligned using MUSCLE v3.8.31 (R. C. Edgar, *Nucleic Acids Res* 32:1792-1797, 2004) in Mesquite software (http://www.mesquiteproject.org). Dot plots to visualize the duplicated coding sequences within the conserved “Cthe_2159” domains were produced using the Dottup tool in EMBOSS v6.5.7 (P. Rice, I. Longden, and A. Bleasby, *Trends Genet* 16:276-277, 2000). Transcriptome reads of *Piromyces finnis* (SRR5487626) were retrieved using the SRA toolkit (R. Leinonen, H. Sugawara, and M. Shumway, *Nucleic Acids Res* 39:2010-2012, 2011) and mapped to the genome assembly using Hisat2 (D. Kim, B. Langmead, and S. L. Salzberg, *Nat Methods* 12:357-360, 2015) (12). Regions of interest containing predicted introns were examined manually with IGV (J. T. Robinson et al., *Nat Biotechnol* 29:24-26, 2011).
