# Supplementary material for: Molecular Dating of the Emergence of Anaerobic Rumen Fungi and the Impact of Laterally Acquired Genes
Source: mSystems. 2019 Aug 27;4(4):e00247-19. doi: 10.1128/mSystems.00247-19 (PMC6712302; doi:10.1128/mSystems.00247-19)
Supplement: FIG S6 [file mSystems.00247-19-sf006.pdf]

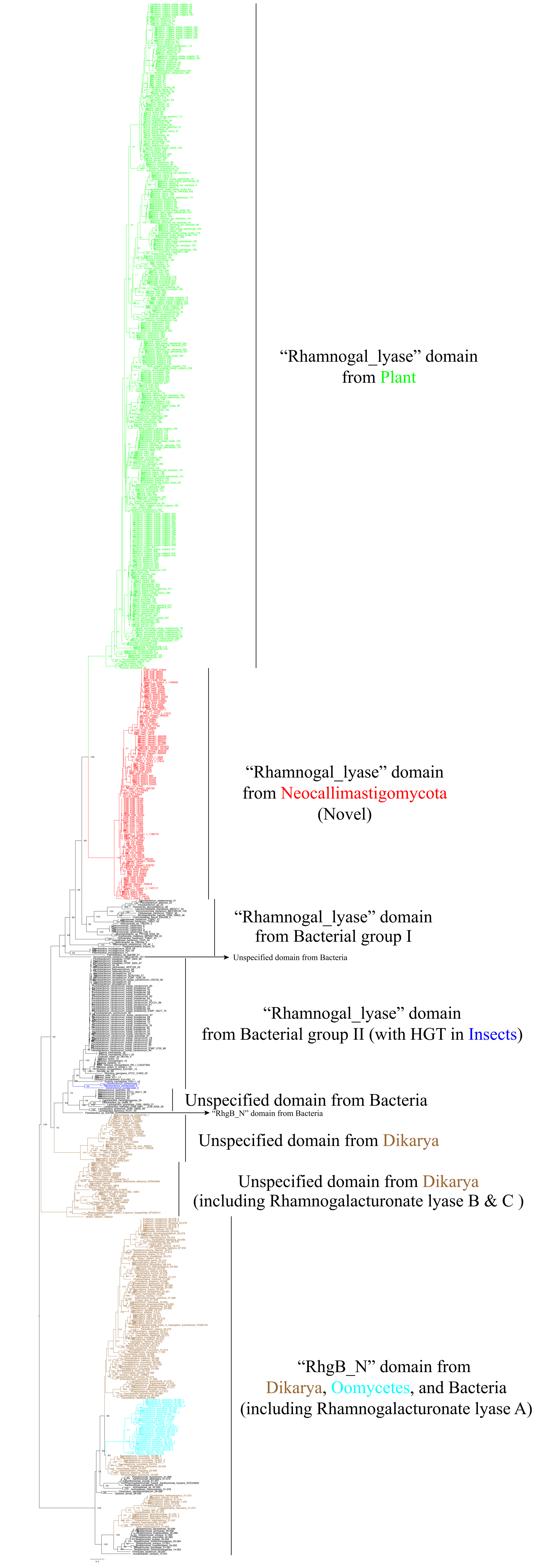

"Rhamnogal_lyase" domain
from Plant
"Rhamnogal_lyase" domain
from Neocallimastigomycota
(Novel)
"Rhamnogal_lyase" domain
from Bacterial group I
Unspecified domain from Bacteria
"Rhamnogal_lyase" domain
from Bacterial group II (with HGT in Insects)
Unspecified domain from Bacteria
"RhgB_N" domain from Bacteria
Unspecified domain from Dikarya
Unspecified domain from Dikarya
(including Rhamnogalacturonate lyase B & C)
"RhgB_N" domain from
Dikarya, Oomycetes, and Bacteria
(including Rhamnogalacturonate lyase A)
