# Supplementary material for: Molecular Dating of the Emergence of Anaerobic Rumen Fungi and the Impact of Laterally Acquired Genes
Source: mSystems. 2019 Aug 27;4(4):e00247-19. doi: 10.1128/mSystems.00247-19 (PMC6712302; doi:10.1128/mSystems.00247-19)
Supplement: TABLE S2 [file mSystems.00247-19-st002.docx]

**Table S2.** Genome information of the animal hosts and diet plants used in the study to infer the genetic elements in Neocallimastigomycota that have a foreign origin

| **Name** | **Taxon** | **Version** | **Source** |
| --- | --- | --- | --- |
| Elephant | *Loxodonta africana* (African savanna elephant) | Loxafr3.0 | (1) |
| Horse | *Equus caballus* | EquCab2.0 | (2) |
| Sheep | *Ovis aries* | Oar_v4.0 | (3) |
| Yak | *Bos mutus* (wild yak) | BosGru_v2.0 | (4) |
| Banana | *Musa acuminata* | DH-Pahang v2 | (5) |
| Palm | *Elaeis guineensis* (African oil palm) | EG5 | (6) |
| Bamboo | *Phyllostachys heterocycla* var. pubescens | v1.0 | (7) |
| Goatgrass | *Aegilops tauschii* subsp. Tauschii | Aet_MR_1.0 | (8) |
| Maize | *Zea mays* | B73 RefGen_v4 | (9) |
| Rice | *Oryza sativa* Japonica Group | Build 4.0 | (10) |
| Brome | *Brachypodium distachyon* | v2.0 | (11) |
| Sorghum | *Sorghum bicolor* | v3 | (12) |
| Arabidopsis | *Arabidopsis thaliana* | TAIR10 | (13) |
| Moss | *Physcomitrella patens* | V1.1 | (14) |

**References**

1. Broad Institute. 2018. Elephant Genome Project.

2. Wade CM, Giulotto E, Sigurdsson S, Zoli M, Gnerre S, Imsland F, Lear TL, Adelson DL, Bailey E, Bellone RR. 2009. Genome sequence, comparative analysis, and population genetics of the domestic horse. Science 326:865–867.

3. The International Sheep Genomics Consortium, Archibald AL, Cockett NE, Dalrymple BP, Faraut T, Kijas JW, Maddox JF, McEwan JC, Hutton Oddy V, Raadsma HW, Wade C, Wang J, Wang W, Xun X. 2010. The sheep genome reference sequence: a work in progress. Anim Genet 41:449–453.

4. Qiu Q, Zhang G, Ma T, Qian W, Ye Z, Cao C, Hu Q, Kim J, Larkin DM, Auvil L, Capitanu B, Ma J, Lewin HA, Qian X, Lang Y, Zhou R, Wang L, Wang K, Xia J, Liao S, Pan S, Lu X, Hou H, Wang Y, Zang X, Yin Y, Ma H, Zhang J, Wang Z, Zhang Y, Zhang D, Yonezawa T, Hasegawa M, Zhong Y, Liu W, Zhang Y, Huang Z, Zhang S, Long R, Yang H, Lenstra JA, Cooper DN, Wu Y, Wang J, Shi P, Wang J, Liu J, Wang J. 2012. The yak genome and adaptation to life at high altitude. Nat Genet 44:946–949.

5. Martin G, Baurens FC, Droc G, Rouard M, Cenci A, Kilian A, Hastie A, Doležel J, Aury JM, Alberti A, Carreel F, D’Hont A. 2016. Improvement of the banana “*Musa acuminata*” reference sequence using NGS data and semi-automated bioinformatics methods. BMC Genomics 17:1–12.

6. Singh R, Ong-Abdullah M, Low ETL, Manaf MAA, Rosli R, Nookiah R, Ooi LCL, Ooi SE, Chan KL, Halim MA, Azizi N, Nagappan J, Bacher B, Lakey N, Smith SW, He D, Hogan M, Budiman MA, Lee EK, Desalle R, Kudrna D, Goicoechea JL, Wing RA, Wilson RK, Fulton RS, Ordway JM, Martienssen RA, Sambanthamurthi R. 2013. Oil palm genome sequence reveals divergence of interfertile species in Old and New worlds. Nature 500:335–339.

7. Peng Z, Lu Y, Li L, Zhao Q, Feng Q, Gao Z, Lu H, Hu T, Yao N, Liu K, Li Y, Fan D, Guo Y, Li W, Lu Y, Weng Q, Zhou C, Zhang L, Huang T, Zhao Y, Zhu C, Liu X, Yang X, Wang T, Miao K, Zhuang C, Cao X, Tang W, Liu G, Liu Y, Chen J, Liu Z, Yuan L, Liu Z, Huang X, Lu T, Fei B, Ning Z, Han B, Jiang Z. 2013. The draft genome of the fast-growing non-timber forest species moso bamboo (*Phyllostachys heterocycla*). Nat Genet 45:456–461.

8. Zimin AV., Puiu D, Luo M-C, Zhu T, Koren S, A.Yorke J, Dvorak J, Salzberg SL. 2017. Hybrid assembly of the large and highly repetitive genome of Aegilops tauschii, a grogenitor of bread wheat, with the MaSuRCA mega-reads algorithm. Genome Res 27:787–792.

9. Jiao Y, Peluso P, Shi J, Liang T, Stitzer MC, Wang B, Campbell MS, Stein JC, Wei X, Chin CS, Guill K, Regulski M, Kumari S, Olson A, Gent J, Schneider KL, Wolfgruber TK, May MR, Springer NM, Antoniou E, McCombie WR, Presting GG, McMullen M, Ross-Ibarra J, Dawe RK, Hastie A, Rank DR, Ware D. 2017. Improved maize reference genome with single-molecule technologies. Nature 546:524–527.

10. The Rice Annotation Project. 2007. Curated genome annotation of *Oryza sativa* ssp. japonica and comparative genome analysis with *Arabidopsis thaliana*. Genome Res 17:175–183.

11. The International Brachypodium Initiative, Vogel JP, Garvin DF, Mockler TC, Schmutz J, Rokhsar D, Bevan MW, Barry K, Lucas S, Harmon-Smith M, Lail K, Tice H, Grimwood J, McKenzie N, Huo N, Gu YQ, Lazo GR, Anderson OD, You FM, Luo MC, Dvorak J, Wright J, Febrer M, Idziak D, Hasterok R, Lindquist E, Wang M, Fox SE, Priest HD, Filichkin SA, Givan SA, Bryant DW, Chang JH, Wu H, Wu W, Hsia AP, Schnable PS, Kalyanaraman A, Barbazuk B, Michael TP, Hazen SP, Bragg JN, Laudencia-Chingcuanco D, Weng Y, Haberer G, Spannagl M, Mayer K, Rattei T, Mitros T, Lee SJ, Rose JKC, Mueller LA, York TL, Wicker T, Buchmann JP, Tanskanen J, Schulman AH, Gundlach H, Beven M, Costa De Oliveira A, Da C. Maia L, Belknap W, Jiang N, Lai J, Zhu L, Ma J, Sun C, Pritham E, Salse J, Murat F, Abrouk M, Bruggmann R, Messing J, Fahlgren N, Sullivan CM, Carrington JC, Chapman EJ, May GD, Zhai J, Ganssmann M, Gurazada SGR, German M, Meyers BC, Green PJ, Tyler L, Wu J, Thomson J, Chen S, Scheller H V., Harholt J, Ulvskov P, Kimbrel JA, Bartley LE, Cao P, Jung KH, Sharma MK, Vega-Sanchez M, Ronald P, Dardick CD, De Bodt S, Verelst W, Inzé D, Heese M, Schnittger A, Yang X, Kalluri UC, Tuskan GA, Hua Z, Vierstra RD, Cui Y, Ouyang S, Sun Q, Liu Z, Yilmaz A, Grotewold E, Sibout R, Hematy K, Mouille G, Höfte H, Micheel T, Pelloux J, O’Connor D, Schnable J, Rowe S, Harmon F, Cass CL, Sedbrook JC, Byrne ME, Walsh S, Higgins J, Li P, Brutnell T, Unver T, Budak H, Belcram H, Charles M, Chalhoub B, Baxter I. 2010. Genome sequencing and analysis of the model grass *Brachypodium distachyon*. Nature 463:763–768.

12. Paterson AH, Bowers JE, Bruggmann R, Dubchak I, Grimwood J, Gundlach H, Haberer G, Hellsten U, Mitros T, Poliakov A, Schmutz J, Spannagl M, Tang H, Wang X, Wicker T, Bharti AK, Chapman J, Feltus FA, Gowik U, Grigoriev I V., Lyons E, Maher CA, Martis M, Narechania A, Otillar RP, Penning BW, Salamov AA, Wang Y, Zhang L, Carpita NC, Freeling M, Gingle AR, Hash CT, Keller B, Klein P, Kresovich S, McCann MC, Ming R, Peterson DG, Mehboob-Ur-Rahman, Ware D, Westhoff P, Mayer KFX, Messing J, Rokhsar DS. 2009. The *Sorghum bicolor* genome and the diversification of grasses. Nature 457:551–556.

13. Swarbreck D, Wilks C, Lamesch P, Berardini TZ, Garcia-Hernandez M, Foerster H, Li D, Meyer T, Muller R, Ploetz L, Radenbaugh A, Singh S, Swing V, Tissier C, Zhang P, Huala E. 2008. The Arabidopsis Information Resource (TAIR): gene structure and function annotation. Nucleic Acids Res 36:D1009–D1014.

14. Rensing SA, Lang D, Zimmer AD, Terry A, Salamov A, Shapiro H, Nishiyama T, Perroud P-F, Lindquist EA, Kamisugi Y, Tanahashi T, Sakakibara K, Fujita T, Oishi K, Shin-I T, Kuroki Y, Toyoda A, Suzuki Y, Hashimoto S, Yamaguchi K, Sugano S, Kohara Y, Fujiyama A, Anterola A, Aoki S, Ashton N, Barbazuk WB, Barker E, Bennetzen JL, Blankenship R, Cho SH, Dutcher SK, Estelle M, Fawcett JA, Gundlach H, Hanada K, Heyl A, Hicks KA, Hughes J, Lohr M, Mayer K, Melkozernov A, Murata T, Nelson DR, Pils B, Prigge M, Reiss B, Renner T, Rombauts S, Rushton PJ, Sanderfoot A, Schween G, Shiu S-H, Stueber K, Theodoulou FL, Tu H, Van de Peer Y, Verrier PJ, Waters E, Wood A, Yang L, Cove D, Cuming AC, Hasebe M, Lucas S, Mishler BD, Reski R, Grigoriev I V, Quatrano RS, Boore JL. 2008. The Physcomitrella genome reveals evolutionary insights into the conquest of land by plants. Science 319:64–69.
